# Evaluation of nutritional status and clinical depression classification using an explainable machine learning method

**DOI:** 10.3389/fnut.2023.1165854

**Published:** 2023-05-09

**Authors:** Payam Hosseinzadeh Kasani, Jung Eun Lee, Chihyun Park, Cheol-Heui Yun, Jae-Won Jang, Sang-Ah Lee

**Affiliations:** ^1^Department of Neurology, Kangwon National University Hospital, Chuncheon, Republic of Korea; ^2^Interdisciplinary Graduate Program in Medical Bigdata Convergence, Kangwon National University, Chuncheon, Republic of Korea; ^3^Department of Computer Science and Engineering, Kangwon National University, Chuncheon, Republic of Korea; ^4^Department of Agricultural Biotechnology, Seoul National University, Seoul, Republic of Korea; ^5^Research Institute of Agriculture and Life Sciences, Seoul National University, Seoul, Republic of Korea; ^6^Department of Neurology, Kangwon National University School of Medicine, Chuncheon, Republic of Korea; ^7^Department of Preventive Medicine, College of Medicine, Kangwon National University, Chuncheon, Republic of Korea

**Keywords:** depression, nutrition, machine learning, classification, interpretability, clinical depression

## Abstract

**Introduction:**

Depression is a prevalent disorder worldwide, with potentially severe implications. It contributes significantly to an increased risk of diseases associated with multiple risk factors. Early accurate diagnosis of depressive symptoms is a critical first step toward management, intervention, and prevention. Various nutritional and dietary compounds have been suggested to be involved in the onset, maintenance, and severity of depressive disorders. Despite the challenges to better understanding the association between nutritional risk factors and the occurrence of depression, assessing the interplay of these markers through supervised machine learning remains to be fully explored.

**Methods:**

This study aimed to determine the ability of machine learning-based decision support methods to identify the presence of depression using publicly available health data from the Korean National Health and Nutrition Examination Survey. Two exploration techniques, namely, uniform manifold approximation and projection and Pearson correlation, were performed for explanatory analysis among datasets. A grid search optimization with cross-validation was performed to fine-tune the models for classifying depression with the highest accuracy. Several performance measures, including accuracy, precision, recall, F1 score, confusion matrix, areas under the precision-recall and receiver operating characteristic curves, and calibration plot, were used to compare classifier performances. We further investigated the importance of the features provided: visualized interpretation using ELI5, partial dependence plots, and local interpretable using model-agnostic explanations and Shapley additive explanation for the prediction at both the population and individual levels.

**Results:**

The best model achieved an accuracy of 86.18% for XGBoost and an area under the curve of 84.96% for the random forest model in original dataset and the XGBoost algorithm with an accuracy of 86.02% and an area under the curve of 85.34% in the quantile-based dataset. The explainable results revealed a complementary observation of the relative changes in feature values, and, thus, the importance of emergent depression risks could be identified.

**Discussion:**

The strength of our approach is the large sample size used for training with a fine-tuned model. The machine learning-based analysis showed that the hyper-tuned model has empirically higher accuracy in classifying patients with depressive disorder, as evidenced by the set of interpretable experiments, and can be an effective solution for disease control.

## 1. Introduction

Depression is a common heterogeneous neuropsychiatric disorder and one of the most common mental disorders associated with high costs, morbidity, and mortality (1, 2). It is characterized by profoundly depressive emotional and cognitive impairment, including sadness (3), loss of self-esteem (4), loss of interest, potentially decreased motivation (5), sleep disturbance, intense feelings of guilt, and difficulty concentrating or making a decision (6, 7). Needless to say that these symptoms adversely affect well-being, life expectancy, and physical and mental health (8, 9). The etiology of depression is multifactorial and dependent on the individual's biological, psychosocial, and social factors (10). These depressive symptoms have a relatively high prevalence and play a crucial role in symptom persistence and recurrence, often making diagnosis and treatment challenging. According to various findings, depression negatively affects over 300 million people worldwide and is estimated to be the leading cause of the disease burden globally by 2030 (11). According to the World Mental Health Survey findings, the lifetime risk of developing depression is higher in high-income countries, with 15% of the population at risk, compared with 11% in low- and middle-income countries (12). Globally, it is estimated that 5% of adults suffer from depression (11). With the rapidly growing number of people with depression worldwide, it is becoming one of the most severe disorders for humankind. In recent years, it has been established that depression affects a significant portion of the population, with a lifetime prevalence rate of 15% (13), endangering people's physical health. It has adversely affected at least 322 million people worldwide and ~4.4% of the world's population, with an incidence rate higher than the rate of global population growth (14). The relationship between nutrition and depression is of growing interest and has attracted considerable attention. To date, accumulating evidence from demographic studies has implicated dietary factors as a substantial risk factor for the development of depression (15–18). The prevalence of depression may be affected by dietary intake patterns, as there is a connection between nutrition and depression (19). A recent study revealed that nutrition plays a pivotal role in the onset, progression, severity, and duration of depression, with poor nutrition contributing to its pathogenesis (20). However, there are only a few published articles on nutrition-based risk factors; hence, further exploration is required to confirm these findings. While identifying depressive disorder in primary care is essential for effective treatment, only approximately half of patients with a depressive disorder are discovered by routine healthcare providers in high-income settings (21). Therefore, it is desirable to identify individuals at risk of the disease at an early stage to enable preventive medical interventions and risk stratification to promote lifestyle modifications as early as possible. This is critical in preventing long-term health complications and fatalities and will help control and manage the disease.

In recent years, technological advances in data science, a multidisciplinary field, have led to a golden opportunity for the healthcare industry to generate a large volume of data from various sources on patient demography, treatment plans, and medical examinations that have led to the compilation of big data. The ever-increasing volume of data created in the healthcare industry requires healthcare professionals to understand how to connect big data for improved efficiency in diagnosis and research operations so that the patient's needs can be addressed (22, 23). Given the various sources of information, knowledge regarding depressive disorders can be mined by automated computerized methods to help clinicians discover hidden patterns in the data. Automated computerized approaches often employ data mining and machine learning (ML) algorithms as computer-aided diagnosis (CAD) systems. Diagnostic decision-making in medical care by clinicians can also be augmented by CAD systems that extract information from clinical features to classify or predict disease. Mining depression-related health data will offer significant opportunities for further discovering clinical information regarding the role of diet variables and facilitate cohort-wide investigations. Data mining is a commonly used technique for processing large amounts of data in the healthcare domain (24, 25). Researchers have applied several data mining and ML techniques to analyze big medical data, helping healthcare professionals predict depression disorders and their risk factors. ML, which is a powerful branch of artificial intelligence (AI), has been widely applied to CAD systems for more robust disease classification and prediction. CAD uses ML methods to analyze patient data and evaluate a patient's condition, which can then be used to assist clinicians in their decision-making process. In the past decade, several CAD techniques have been developed to analyze risk factor and predictor relationships (26–28).

Efficient early detection of depressive disorder is crucial in managing mental health at a population level. The widespread adoption of electronic health records (EHRs) has facilitated the analysis of large datasets to investigate clinical questions using computational analysis. A systematic electronic medical records diagnosis of depression was found to be an effective method for diagnosing clinical depression with the area under the curve (AUC) of 0.77% (29) as a baseline prediction rate. A study by Nam et al. (30), using the extreme gradient boosting (XGBoost) machine learning classifier, aimed to identify essential depression-associated factors from the K-NHANES dataset with 120 variables for 12,596 cases and achieved an 86% ROC curve score after feature selection. This study, however, suffered from a highly imbalanced problem with a 2.7% prevalence of depression. To detect depression in the K-NHANES dataset, Oh et al. used customized deep neural network and machine learning in which logistic regression obtained the highest performance with AUC of 0.77% (31). However, there was a severe imbalance problem in which there were only 344 cases (7.0%) of depression out of a total of 4,949 cases.

Collectively, while this investigation aims to further elucidate the feasibility of CAD-based analysis for depression diagnosis, additional exploration is required to support the current existing findings based on the direct impact of nutritional factors. There were several critical issues while using many variables yet to be addressed ranging from difficulty in interpretation, increased computational complexity, cost and time constraints when collecting data, overfitting, multicollinearity, and redundancy (32, 33). In addition to the insightful ML-based studies with strong diagnostic accuracy, there have been limited efforts to create an interpretable and explainable method for predicting depression. Furthermore, the nutrition-related risk factors of depression have not been fully explored in an ML-based approach in an explainable manner with an individual and the global level that contributed to the prediction. This study aimed to develop an interpretable data-driven CAD approach capable of distinguishing participants with and without depression using clinical and nutrition-related variables and data from the K-NHANES. We constructed and compared depression prediction models using supervised ML algorithms: logistic regression (LR), support vector machine (SVM), decision tree (DT), random forest (RF), and extreme gradient boosting (XGB). This study used model optimization to select a suitable hyperparameter for the pre-trained model on the depression classification task. We also applied feature-importance techniques using permutations to determine the relative importance of these features. To aid physicians in decision-making, we present a local interpretable approach that offers relevant and actionable information. Furthermore, we consider this a significant advancement in the use of ML to study clinical depression. Our method not only dramatically increases the ability to diagnose depression at an early stage but also explains the predictions from accurate and complex models to understand the causes of the prognosis for critical intervention methods to be designed. The major contributions of this study are as follows. First, we prepared a cohort database containing accessible clinical and standard nutritional variables. These variables were collected from K-NHANES, a large-scale database. Second, we employed several well-known ML models and trained the models from scratch using the two collected datasets. The models showed significant performance in the classification of depression. Third, we performed a comparative explainable ML analysis and visualized the interpretation using several interpretability techniques. We believe that our work is a crucial step toward advancing the understanding and credibility of trustworthy precision medicine by incorporating a comprehensive list of explanations for depression prediction at both the local and global levels.

The rest of this article is organized as follows: Section 2 provides a detailed description of the materials and methods, and Section 3 describes the experimental data and results. Section 4 provides a discussion. Finally, Section 5 presents the conclusion and proposes future research directions.

## 2. Materials and methods

### 2.1. Dataset description

In this study, the K-NHANES dataset (34), a dataset from a large-scale cross-sectional study, was used to investigate the nutritional risk factors for depression. The K-NHANES is a longitudinal survey initiated in 1998 designed to assess the health and nutritional status of people living in Korea. It provides researchers with vital information to determine the causes of the disease based on the population's distribution of health problems and risk factors. This survey is conducted annually by the Korea Disease Control and Prevention Agency (KDCA). The KNHANES dataset includes demographic, socioeconomic, comorbidities, and dietary features. According to the clinical professional and research question, 4,804 cases meeting the inclusion criteria of 33 variables were identified as predictors for depression classification analyses. All participants were classified as depressed or non-depressed. The non-depression class label was assigned to 4,031 samples (84% of the dataset), while the depression class label was assigned to 773 samples (16% of the dataset).

Nutrition-related markers included energy, water, protein, fat, carbohydrates, and fiber. The scores on the nutritional variables objectively show the participants' eating habits. For comparison, we generated two datasets, each with 27 variables. One dataset was used for the original values, and the second dataset was used for quantile-based values for nutritional features. The numeric input variables may have a non-standard or highly skewed distribution. Outliers in the data, multimodal distributions, highly exponential distributions, and other possibilities might be responsible for this anomaly. In this case, the raw nutritional scores for each participant were converted into quantiles. The details and descriptions of these features are listed in Table 1. All K-NHANES data, except the pediatric survey information, are in the public domain and are available at the National Center for Health Statistics (https://www.cdc.gov/nchs/nhanes).

**Table 1:** Characteristics of depression patients datasets.

**No**.	**Variable name**	**Non-depression *N =* 4,031**	**Depression *N =* 773**	**OR (95% CI)**	***P-value***	**Abbreviation**
**1**	**Gender**					
	Female	2,204 (54.68%)^a^	516 (66.75%)	1.55 (1.30–1.83)		
**2**	**Age**	53.20 ± 17.24	49.38 ± 17.51	0.98 (0.98–0.99)	**<0.001**	
**3**	**Income**				**<0.001**	INCM
	High	1,050 (26.05%)	153 (19.79%)	1.00		
	Middle low	1,022 (25.35%)	196 (25.36%)	1.32 (1.04–1.68)		
	Middle high	1,013 (25.13%)	172 (22.25%)	1.30 (0.97–1.74)		
	Low	946 (23.47%)	252 (32.60%)	1.85 (1.42–2.41)		
**4**	**Education level**				**0.003**	EDULV
	No school	71 (1.76%)	26 (3.36%)	2.37 (1.35–4.14)		
	Elementary school	421 (10.44%)	105 (13.58%)	1.36 (0.85–2.18)		
	Middle school	360 (8.93%)	80 (10.35%)	1.25 (0.76–2.05)		
	High school	1,529 (37.93%)	224 (28.98%)	1.18 (0.77–1.81)		
	2/3 Years college	453 (11.24%)	113 (14.62%)	1.65 (1.08–2.52)		
	College	962 (23.87%)	190 (24.58%)	1.24 (0.78–1.97)		
	Graduate school	233 (5.78%)	35 (4.53%)	1.00		
**5**	**Occupation**					OCCU
	No	2,706 (67.13%)	367 (47.48%)	1.95 (1.64–2.32)		
**6**	**Marriage**					
	Yes	3,330 (82.61%)	562 (72.70%)	1.00		MARRY
	No	701 (17.39%)	211 (27.30%)	1.93 (1.56–2.38)		
**7**	**Economic status**					ECONSTAT
	Yes	2,706 (67.13%)	367 (47.48%)	0.51 (0.43–0.61)		
**8**	**Body mass index**	24.02 ± 3.50	24.27 ± 4.17	1.00 (0.98–1.03)	**0.897**	BMI
**9**	**Quality of life**	0.97 ± 0.08	0.88 ± 0.16	4.46 (3.63–5.47)	**<0.001**	QOL
**10**	**Family history**					FHIS
	Yes	2,621 (65.02%)	516 (66.75%)	1.29 (1.07–1.56)		
**11**	**Exercise**					
	Yes	3,490 (86.58%)	634 (82.02%)	0.77 (0.59–1.00)		EXC
**12**	**Drinking**					
	Yes	3,542 (87.87%)	686 (88.75%)	1.27 (0.98–1.66)		DRK
**13**	**Smoking**					
	No	2,590 (64.25%)	466 (60.28%)	1.00		SMK
	Past	885 (21.95%)	134 (17.34%)	0.86 (0.68–1.11)		
	Current	556 (13.79%)	173 (22.38%)	1.61 (1.26–2.04)		
**14**	**Sleeptime wk**.	7.01 ± 3.29	6.34 ± 3.75	0.75 (0.71–0.81)	**<0.001**	SLPWK
**15**	**Sleeptime wd**.	7.53 ± 3.36	7.01 ± 3.94	0.89 (0.83–0.94)	**<0.001**	SLPWD
**16**	**Self-recognition stress**				**<0.001**	SRS
	Feels little bit	2,574 (63.86%)	278 (35.96%)	0.06 (0.04–0.09)		
	Not little	695 (17.24%)	32 (4.14%)	0.02 (0.01–0.03)		
	Feels a lot	676 (16.77%)	326 (42.17%)	0.25 (0.17–0.37)		
	Feels very much	86 (2.13%)	137 (17.72%)	1.00		
**17**	**Stress recognition rate**				**<0.001**	SRR
	Less stress	3,269 (81.10%)	310 (40.10%)	1.00		
	High stress	762 (18.90%)	463 (59.90%)	6.20 (5.19–7.40)		
**18**	**Suicide attempt**					
	No	4,024 (99.83%)	755 (97.67%)	0.07 (0.03–0.17)		SUA
**19**	**Health status**				**<0.001**	HS
	Bad	2,910 (72.19%)	688 (89.00%)	1.00		
	Middle	946 (23.47%)	73 (9.44%)	0.31 (0.24–0.42)		
	Good	175 (4.34%)	12 (1.55%)	0.25 (0.12–0.52)		
**20**	**Chronic disease**					CHRD
	No	2,507 (62.19%)	429 (55.50%)	0.87 (0.72–1.04)		
**21**	**Cancer**					
	No	3,953 (98.06%)	755 (97.67%)	0.97 (0.55–1.72)		
**22**	**Energy**	1,824.55 ± 817.05	1,744.28 ± 923.94	1.00 (1.00–1.00)	**0.057**	
**23**	**Water**	960.30 ± 559.66	904.21 ± 589.02	1.00 (1.00–1.00)	**0.185**	
**24**	**Protein**	68.19 ± 38.55	62.90 ± 35.67	1.00 (0.99–1.00)	**0.013**	
**25**	**FAT**	44.82 ± 35.18	42.80 ± 34.66	1.00 (1.00–1.00)	**0.269**	
**26**	**Carbohydrate**	266.93 ± 107.17	256.33 ± 118.06	1.00 (1.00–1.00)	**0.103**	CRBHDR
**27**	**Fiber**	24.79 ± 13.24	22.77 ± 14.55	0.99 (0.98–1.00)	**0.019**	
**28**	**Energy quintile**	2.02 ± 1.41	1.88 ± 1.42	0.80 (0.60–1.05)	**0.104**	QENERGY
**29**	**Water quintile**	2.03 ± 1.40	1.85 ± 1.47	0.78 (0.58–1.06)	**0.116**	QWATER
**30**	**Protein quintile**	2.03 ± 1.41	1.86 ± 1.43	0.73 (0.55–0.99)	**0.042**	QPROTN
**31**	**FAT quintile**	2.01 ± 1.42	1.95 ± 1.40	0.99 (0.72–1.36)	**0.964**	QFAT
**32**	**Carbohydrate quintile**	2.02 ± 1.40	1.88 ± 1.46	0.74 (0.58–0.94)	**0.016**	QCARBO
**33**	**Fiber quintile**	2.05 ± 1.40	1.76 ± 1.45	0.61 (0.47–0.79)	**0.002**	QFIBER

a Data are mean ± SD values except were indicated otherwise; n/N (%), Figures in parentheses are percentages.

Bold values denote statistically significant at *p* < 0.001.

### 2.2. Pre-processing

Prior information regarding the raw data directly affects the performance of the optimized classifier. In this regard, pre-processing the data is crucial to obtaining efficient classification performance before evaluating the data using ML algorithms (35). Among various data preprocessing methods, data normalization is an essential preprocessing step in which the data are either scaled or transformed to minimize the bias of those features whose numerical contribution is higher in discriminating pattern classes. It also helps in determining the operational speed of the model. Before the modeling implementation, preprocessing was conducted in the form of missing value replacement and data normalization.

### 2.3. Machine learning model development

**Figure 1:**
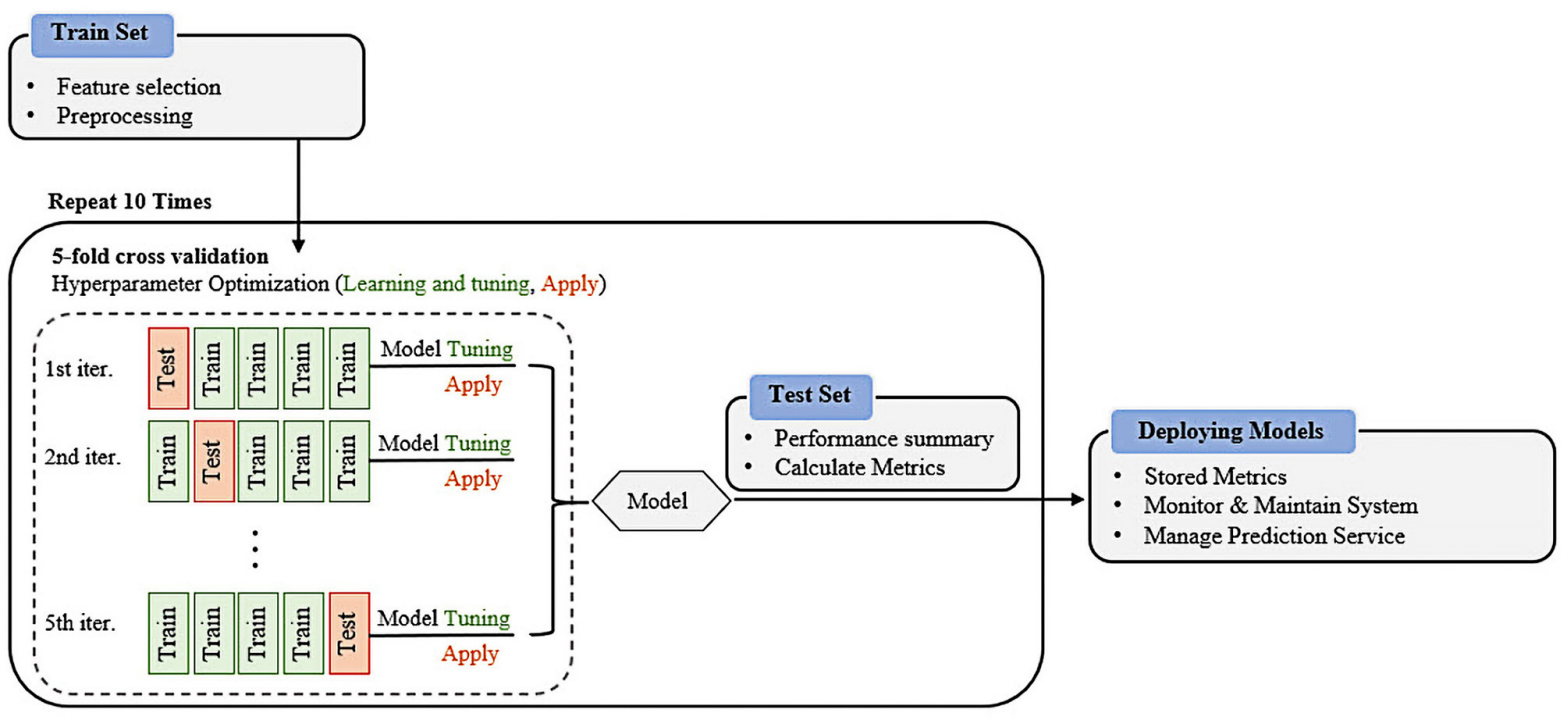
Overview of the machine learning workflows for prediction of depressive disorder.

This study examined the performance of five widely used and well-known ML classifiers: LR, SVM, RF, DT, and XGB. These algorithms were selected because of their apparent inductive tendencies and capacity to explore complex relationships between variables, including nonlinear patterns. A cross-validation scheme was applied to avoid potential overfitting problems (36). For this, a 5-fold cross-validation was performed with the training dataset as inner cross-validation for hyperparameter determination (37) and outer cross-validation performance evaluations. We randomly split the K-NHANES dataset into two parts: 70% as the training dataset and 30% as the test dataset for internal validation. A grid search with cross-validation (GridSearchCV) was used to fine-tune each model to increase the model efficiency and obtain the best possible result. The classifiers were trained using different combinations of parameters. The training dataset (70% of the entire dataset) was randomly split into k (5) stratified folds that preserved the relative proportion of the two classes (non-depression and depression). The k−1 subset was used for training, and one subset was used for testing. Each fold was utilized once as a test set before we moved on to the next, whereas the other remaining folds were temporarily combined to create a training set for model creation. This was repeated 10 times to use all possible training and test set combinations. Optimal hyperparameters were determined according to the best area under the curve (AUC) in the validation set (see Supplementary Table 1 for selected hyperparameters). Finally, the performance of the models was evaluated on the holdout testing set using the tuned hyperparameters. The test set provided independent validation, demonstrating the model's ability to generalize unseen data. This scenario allows all data points to be classified and validated while maintaining a separate training set. A schematic graphic overview of the work process of grid search with cross-validation of the proposed supervised machine learning formulas for disease detection is shown in Figure 1.

#### 2.3.1. Logistic regression

Logistic regression (LR), another technique from the field of statistics borrowed by ML, is the process of modeling the probability of a discrete outcome given an input variable (38). The outcomes were measured using a dichotomous variable. LR is a transformation of linear regression using the sigmoid function, which obtains a linear combination of variables and then applies them to a nonlinear sigmoidal function. LR is a valuable analysis method for classification problems rather than a regression model, and it attempts to achieve reliable performance with linearly separable classes and can also be generalized to multiclass classification.

#### 2.3.2. Random forest

Random forest (RF) is a supervised ML algorithm that is widely used in classification and regression tasks and has recently been applied to engineering practice (39). It is also one of the most widely used algorithms owing to its simplicity and diversity. It uses ensemble learning, which constructs a set of classifiers, instead of one classifier, to provide solutions to complex problems. Each node tests a particular feature, and the tree leaves represent the output labels. The final result is obtained by aggregating the outputs from all the leaves, which is a powerful technique that combines many classifiers to solve complex problems. It consists of many individual decision trees that operate as ensembles. Each tree in the RF gives a class prediction, and the class with the most votes becomes the prediction of our model.

#### 2.3.3. Decision trees

Decision trees (DTs) are one of the most used machine learning algorithms. They are used for both classification and regression (40). A DT is a support tool with a tree-like structure that models the probable outcomes and possible consequences by providing conditional control statements. They include branches representing the decision-making steps that can lead to favorable results. In the generated decision tree, each leaf node represents a class label of the target variable, and each internal node corresponds to a feature at each stage. The constructed DT is a binary tree built using a classification algorithm called classification and regression trees (CART). DTs are among the best learning algorithms based on various learning methods. They boost predictive models in terms of accuracy, ease of interpretation, and stability. These tools are also effective in fitting non-linear relationships because they can solve data-fitting challenges, such as regression and classification.

#### 2.3.4. Support vector machine

This method involves determining the class of data points by appropriate hyperplanes in a multidimensional space (41). Using SVM, we aim to find a hyperplane that separates cases of two categories of variables that take up neighboring clusters of vectors, one on one side and the other on the other side. The support vectors are those that are closer to the hyperplane. The training and test data were used in the SVM. The training data are broken up into target values and attributes, and SVM produces a model for predicting the target values for the test data.

#### 2.3.5. XGBoost

XGBoost is a DT-based ensemble ML algorithm that uses a gradient-boosting framework (42). It is also one of the most commonly used algorithms for prediction problems involving unstructured data (images, text, etc.). Artificial neural networks tend to outperform all other algorithms and frameworks. However, when it comes to small-to-medium structured/tabular data, DT-based algorithms are now considered the best in class.

#### 2.3.6. Evaluation metrics

To evaluate the performance of a binary classification model, selecting the appropriate metrics based on the requirements of the user is highly important. The performance of the ML models was comprehensively measured using different widely used metrics, including accuracy (ACC), recall (REC), precision (PREC), F1 score, the area under the receiver operating characteristic curve (AUROC), and the area under the precision-recall curve (AUPRC). Predictive values were also demonstrated in a two-by-two confusion matrix. For the metric definitions, we used the following abbreviations: true positive (TP), true negative (TN), false positive (FP), and false negative (FN), and then calculated the corresponding values for each metric. A TP is a positive depression outcome in which the model correctly predicts the depression class. Similarly, a TN is an outcome in which the model correctly predicts the non-depressive class. An FP is an outcome in which the model incorrectly predicts a non-depression class as a depression class. An FN is an outcome in which the model incorrectly predicts a depression case as a non-depression case. The equations for these metrics are as follows.

Accuracy (ACC) is the ratio of the overall correctly predicted samples to the total number of examples in the evaluation dataset.


$$\begin{array}{l} ACC=\frac{Correctly\;classifieds\;samples}{All\;samples}=\frac{TP+TN}{TP+FP+TN+FN}\; \end{array}$$

Recall (REC), also known as the sensitivity or true positive rate (TPR), is the ratio of correctly predicted positive cases from all samples assigned to the actual positive cases.


$$\begin{array}{l} REC=\frac{True\;positive\;samples}{samples\;classified\;positive}=\frac{TP}{TP+FN}\; \end{array}$$

Precision (PREC) is the ratio between correctly predicted positive samples in all samples assigned to the positive class.


$$\begin{array}{l} PREC=\frac{Samples\;correctly\;classified}{samples\;assigned\;to\;class}=\frac{TP}{TP+FP}\; \end{array}$$

F1 score (F1) is generally defined as the harmonic mean of precision and recall, which penalizes extreme values of either.


$$\begin{array}{l} F1=2\times \frac{precision\;\times \;recall\;}{precision\;\times \;recall\;}=\frac{2\times TP}{2\times TP+FP+FN}\; \end{array}$$

The receiver operating characteristic (ROC) curve is a valuable metric showing the performance of a classification model at all classification thresholds (43). It is widely used in binary classification and has two parameters. The area under the precision-recall curve is a valuable metric for classifying imbalanced data (44). We took advantage of the Eli5 and PDP for global and local interpretable model-agnostic explanations (LIME) and SHapley Additive exPlanations (SHAP) libraries for local explainability to make the “Black Box” ML models explainable by assigning weights to different features, which signifies their importance in classification.

### 2.4. Statistical analysis

The programming work for this study was performed in Python programming language (version 3.9) (45). All data pre-processing and analysis were carried out using Pandas (46), NumPy (47), Python libraries for data manipulation and analysis, and Scikit-learn (48), a Python module integrating a wide range of ML algorithms. We performed all analyses on 24 cores of an Intel(R) Xeon(R) Gold 5118 CPU @ 2.30 GHz, RAM 128 GB (Intel Corporation, Santa Clara, CA, USA) running Windows 10 Pro.

## 3. Results

### 3.1. Data description

The general characteristics of the study population are summarized in Table 1. The study population comprised 2,084 men (40.19%) and 2,720 women (59.81%). Compared with individuals with depression, participants without depression tended to be older (53.20 ± 17.51 and 53.20 ± 17.24 years, respectively). Regarding stress-related variables, the depression group had a higher percentage (59.90%) of high stress than the non-depression group. Regarding sleep markers, participants with depression had lower scores than the non-depression group, and this trend was the same for income and quality-of-life variables. Nutrient dietary variables were found to be statistically different between the groups, with the depression group showing slightly lower scores than the non-depression group. Further details are provided in Table 1.

### 3.2. Data exploration

**Figure 2:**
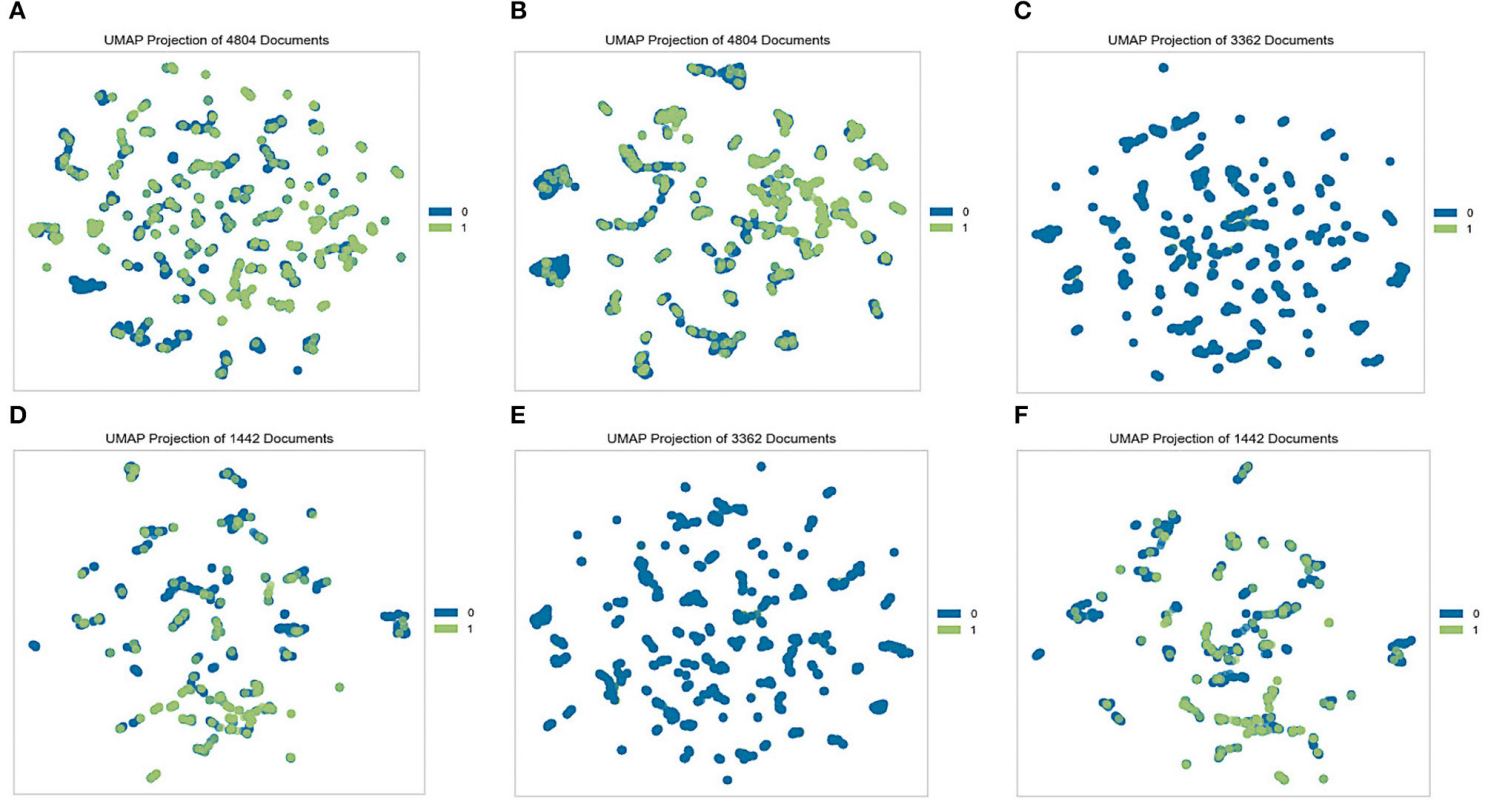
Visualization of non-depression and depression space with the UMAP method. Each dot represents a patient in a two-dimensional space, and its color represents the group. Blue dots (0) represent non-depression people and green dots (1) represent patients' depression. **(A)** Original dataset, **(B)** quantile-based dataset, **(C)** test set from the original dataset, **(D)** train set from the original dataset, **(E)** test set from the quantile-based dataset, and **(F)** train set from the quantile-based dataset.

When dealing with data, an exploratory analysis must be performed. At the outset of each data analysis process, investigators engage in “data exploration,” during which they characterize datasets via data visualization and statistical methods (49). Exploratory data analysis techniques such as clustering and correlation can be used to identify the underlying structure and fundamental correlations between variables in the data. Data visualization is an essential element in data exploration analysis (50). To effectively explore the two datasets generated from the K-NHANES database, two well-known analyses were performed: a population distribution conducted by the uniform manifold approximation and projection (UMAP) technique (51) and the Pearson correlation analysis between variables (52). In this study, we sought to quantitatively compare the K-NHANES database population frequencies determined by UMAP-guided dot-plot-based visualizations. The ability of this approach to accurately define population space distribution is vital for establishing its general validity. Figure 2 depicted a graphical representation of the K-NHANES database sample in the two-dimensional space projected by UMAP. The results demonstrate the full complexity of depression diagnostics, as illustrated by visualizing the outcome space of non-depression and depression from our data using the UMAP method (Figure 2).

Analyzing correlations is a vital step in data analysis and ML tasks. It allows data scientists to understand the possible patterns and connections between two variables or a group of variables and helps in choosing better models (53). This method is widely applied in medical analysis (54). To further analyze the collected variables in the datasets, a heat map of the Pearson correlation coefficients between the variables was constructed (Supplementary Figure 1). Pearson correlation coefficient helps to represent the relationship between two and/or a group of features (52). It also measures the strength of the correlation between variables together. The obtained results show that all variables had a good range of correlation and the dataset was free of multicollinearity, with only a few variables having a slightly strong correlation with each other. Two stress-related variables have a highly negative correlation value. The same pattern also concerns the correlations between marriage and age, smoking, and gender. All these variables are essential parameters in depression disease occurrences (55–57). Additionally, this synergic relationship might help increase the accuracy of the machine learning prediction rate. Chronic disease was also found to have a positive correlation with age, which has been highlighted in different studies (58, 59). All nutrition-related features have a positive correlation together. Variable correlation is further illustrated in Supplementary Figure 1.

**Figure 3:**
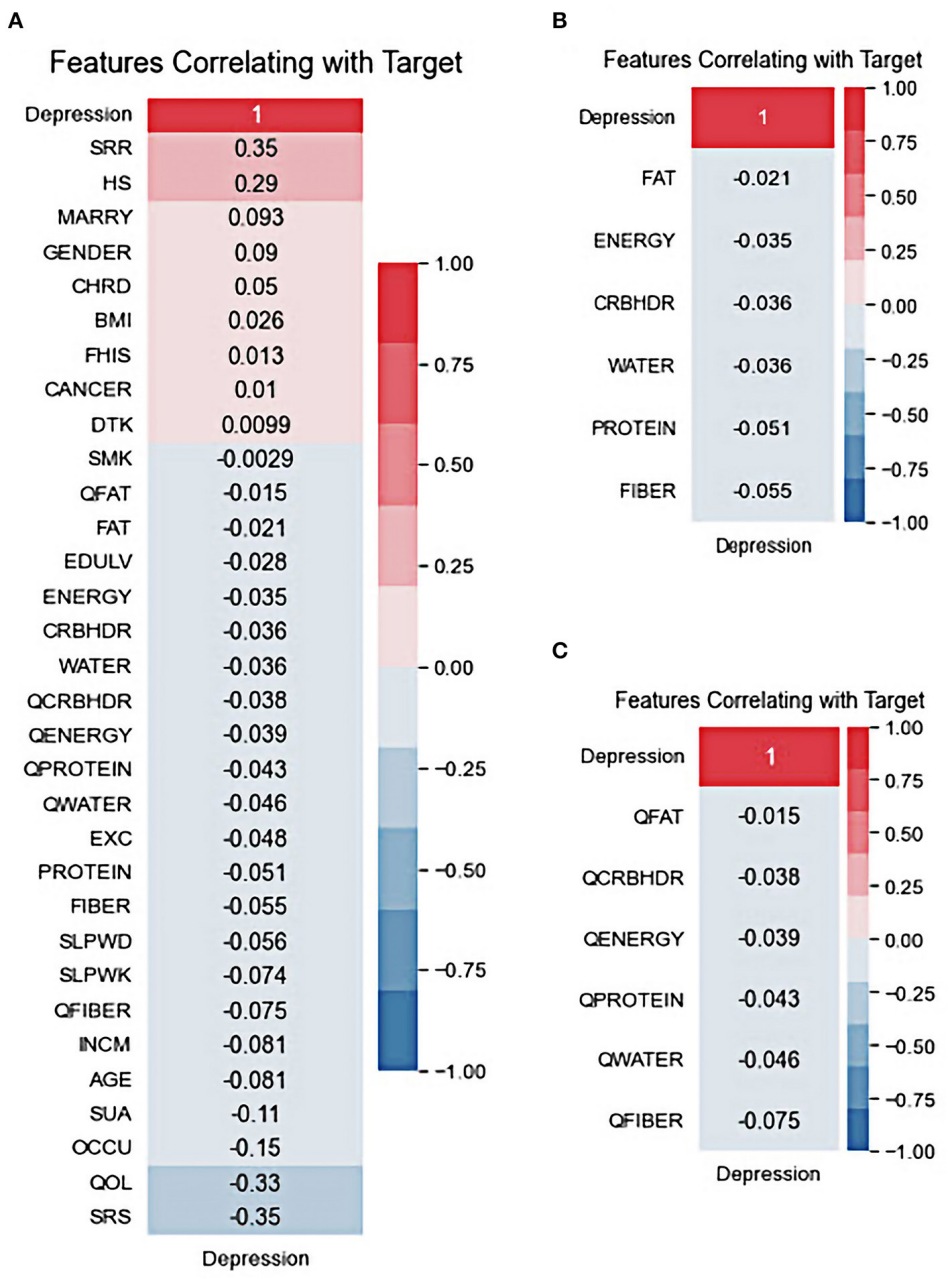
Features correlating with target: **(A)** All variables, **(B)** nutritional default variables, and **(C)** nutritional quantile-based variables.

The correlation between the predictor variables and target outcomes was also investigated. Figure 3 shows the Pearson correlation coefficients for the target variables. It was demonstrated that the low-stress recognition rate, in a positive way, and the self-recognition of stress, in a negative way, have the highest correlation with depression outcomes. In addition, health status and life quality showed strong positive and negative correlations of at least 29 and 33, respectively, with the target outcome. Drinking, smoking, fat quantiles, and fat intake also showed a lower correlation with the target outcomes. Although drinking is positively correlated with patient outcomes, controversy exists over the drinking variable, which is negatively related to depression outcomes. All nutrition variables were negatively correlated with depression; however, among them, fiber quantile, fiber, and protein were the top three highly correlated diet-based features with depression. This is in line with the results of other reports in which the low protein intake groups had a significantly higher risk of depression than the standard protein intake groups (60–62). Several studies have demonstrated a potential relationship between depression and dietary fiber intake, in which an increased intake of total dietary fiber is associated with a lower likelihood of depression (63, 64).

### 3.3. Comparison of model performance

Table 2 presents the comparison results of the ML algorithms on the K-NHANES datasets (original and quantile-based). In the original dataset, the results indicated that among all classification models, except the DT algorithm, all models demonstrated high predictive performance, with AUCs ranging between 81 and 85%. The RF model achieved the highest AUC (84.96%), followed by the LR (84.22%) and the XGB (84.17%). However, the DT was the worse classifier, with 69.77% AUC. There were significant differences between the classification performance of the algorithms (one-way ANOVA; *F* = 100.45; *p* < 0.001). Regarding accuracy, XGB predicted depression with the highest accuracy (86.18%), followed by the RF (84.76%) and the SVM (79.50%). The algorithms' classification performance exhibited noteworthy variations (one-way ANOVA; *F* = 51.56; *p* < 0.001).

**Table 2:** Performance measures analysis for the different machine learning models.

**Models**	**Accuracy**	**Precision**	**Recall**	**F1-score**	**AUC**
**Original**					
DT	75.14 (74.19, 76.09)	35.03 (33.61, 36.46)	68.45 (66.06, 70.83)	46.03 (44.67, 47.39)	69.76 (67.14, 72.37)
LR	78.64 (77.89, 79.39)	41.15 (39.26, 43.03)	75.95 (74.25, 77.66)	53.00 (51.40, 54.59)	84.22 (80.24, 88.19)
XGB	86.18 (85.59, 79.39)	61.39 (58.73, 43.03)	38.40 (36.07, 77.66)	46.53 (44.60, 54.59)	84.17 (80.89, 88.19)
RF	84.76 (84.21, 85.30)	52.29 (49.95, 54.63)	52.75 (50.30, 55.21)	51.69 (50.04, 53.34)	84.96 (80.31, 89.61)
SVM	79.50 (78.94, 80.07)	40.58 (38.97, 42.19)	60.41 (58.33, 62.49)	48.18 (46.80, 49.56)	80.40 (75.33, 85.47)
**Quantile**					
DT	75.13 (74.22, 76.04)	36.26 (35.01, 37.52)	70.71 (68.66, 72.75)	47.98 (46.75, 49.20)	74.97 (68.28, 81.67)
LR	78.35 (77.67, 79.03)	40.60 (38.92, 42.29)	75.23 (73.56, 76.90)	52.40 (50.95, 53.84)	84.03 (80.13, 87.93)
XGB	86.02 (85.44, 79.03)	59.71 (57.43, 42.29)	40.30 (38.07, 76.90)	47.49 (45.79, 53.84)	85.34 (82.36, 87.93)
RF	84.02 (83.45, 84.58)	50.14 (47.87, 52.41)	59.14 (56.60, 61.68)	53.85 (52.49, 55.21)	85.30 (80.92, 89.69)
SVM	79.67 (79.17, 80.18)	39.60 (37.90, 41.30)	51.02 (48.75, 53.28)	44.10 (42.75, 45.46)	77.53 (74.14, 80.92)

Values reported as mean (95% confidence interval).

AUC, Area under the ROC Curve; DT, Decision Tree; LR, Logistic Regression; XGB, Extreme Gradient Boosting; RF, Random Forest; SVM, Support Vector Machine.

In the presence of imbalanced classes, accuracy metrics will not provide sufficient performance measures. Therefore, in addition to AUC, the harmonic mean of recall and precision (F1 score) was applied as an additional performance measure for the selected ML algorithms. The F1 score obtained by LR (53%) was the best in predicting depression, followed by the RF (51.69%) and the SVM (48.18%). Simultaneously, the DT model reported the lowest performance for depression classification, with a 46.03% F1 score. There were significant differences between the classification performance of the algorithms (one-way ANOVA; *F* = 11.73; *p* < 0.001). The XGB classifier achieved the highest precision rate (61.39%), and the LR model with the best recall score (75.95%). The AUC performance of the quantile-based nutrition features values indicated a slight increase compared to the original nutrition features values. The XGB algorithm obtained the most accurate prediction for depression with an AUC of 85.34%. The RF algorithm came second with a prediction AUC rate of 85.30%, followed by the LR algorithm with an AUC rate of 84.03%. There were significant differences between the classification performance of the algorithms (one-way ANOVA; *F* = 90.13; *p* < 0.001).

Model performances measured by accuracy revealed the XGB classifier as the best model with 86.02% accuracy, followed by RF with 84.02% accuracy. There were significant differences between the classification performance of the algorithms (one-way ANOVA; *F* = 40.44; *p* < 0.001) (see Supplementary Table 2 for more details). Additionally, the XGB classifier achieved the highest precision rate (59.71%), the LR model with the best recall score (75.23%), and RF achieved the best F1 score (53.85%). Overall, the results seen in the quantile-based datasets indicate that they may have the potential for use in research investigations. However, only accuracy metric performance showed a slight decrease when using quantile-based nutrition features values compared to original values. The LR model's recall was best among all models in either quantile-based or original nutrition datasets.

In ML, the learning curve is a widely used diagnostic tool to demonstrate how well a model will perform in response to changes in the number of training samples. These curves plot the training and test performances of a sample of training examples by incrementally adding new training examples. It is possible to determine whether more training instances result in a higher validation score using the learning curves. Additionally, this metric can be used to diagnose underfit, overfit, or good fit models. The learning curve of the training and test accuracy for different sizes of data points is shown in Supplementary Figure 2. It can be observed that the training and test performances converged for an increasing number of training samples. The graph generated by XGBoost indicates that the predictor is somehow overfitted because of the increasing gap between the training and test curves with a similar example used for both datasets. As soon as the training metrics improve, the validation metrics worsen. For the SVM models, the test metrics were slightly enhanced as soon as the training metrics worsened. Based on the plot, LR performed better with increasing samples in all cases. Generally, the narrower the gap, the lower the variance. The addition of new training instances to the RF model is likely to lead to better models. The validation curve did not plateau at the maximum training set size. It still has the potential to decrease and converge toward the training curve, similar to the convergence observed in the linear regression case.

**Figure 4:**
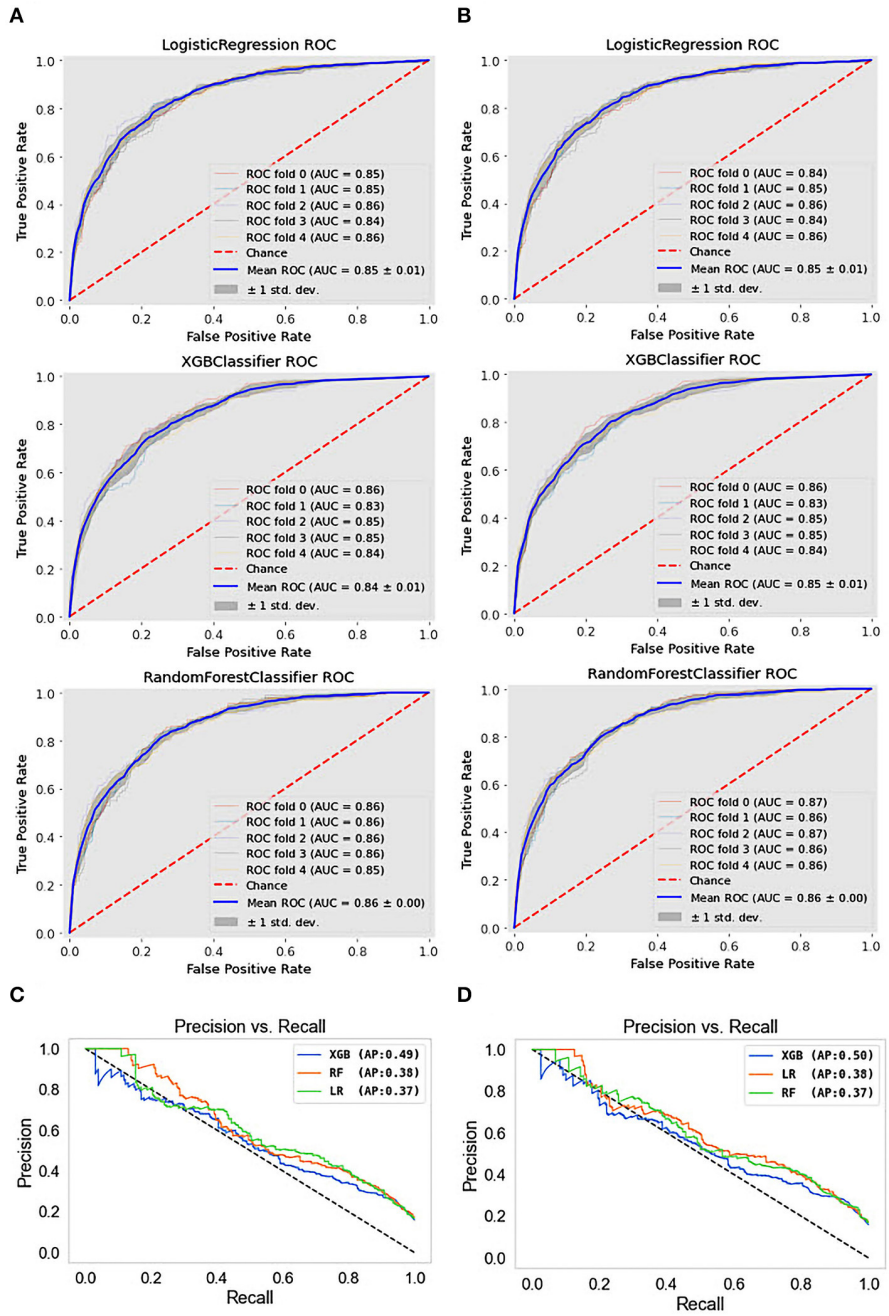
ROC curves and precision-recall PRC curve of top three machine learning models. **(A)** ROC curves for the original dataset, **(B)** ROC curves for the quantile-based dataset, **(C)** PRC curves for the original dataset, and **(D)** PRC curves for the quantile-based dataset.

The results obtained from the predictive models were further investigated using receiver operating characteristic (ROC) and precision-recall (PR) curves. The ROC curve shows the relationship between the rates of TP and FP. The ROC curves for the different types of depression ML classifications are presented in Figures 4A, B. This visualization revealed that the RF model for depression prediction performed better, with an area under the curve (AUC) of 86%. This indicates that the model had more TPs and fewer FPs. In contrast, the worst performance was exhibited by the SVM and DT models for depression disorder, with an AUC of 81%. We can see that the AUC values are similar when we use a dataset with quantile-based variables. PR curves and the areas under them are widely used to summarize the performance of ML classifier results, particularly when evaluating classifiers on imbalanced datasets. This curve represents the tradeoff between the proportion of positively labeled examples that are truly positive (precision) and the ratio of correctly classified positives (recall). Although they have been successfully applied to a wide range of medical problems and have demonstrated significant advantages in recent years (65, 66), their strength in depression disorders has so far been mostly unexplored. When comparing the prediction performance using the PRC-AUC (Figures 4C, D), the XGB classifier demonstrated a higher ability to distinguish patients with depression (minority class) than the RF and LR classifiers with 49 and 50% in original and quantile-based databases, respectively. The RF model represents the second-best model in the original database (39%) and LR in the quantile-based dataset (38%).

**Figure 5:**
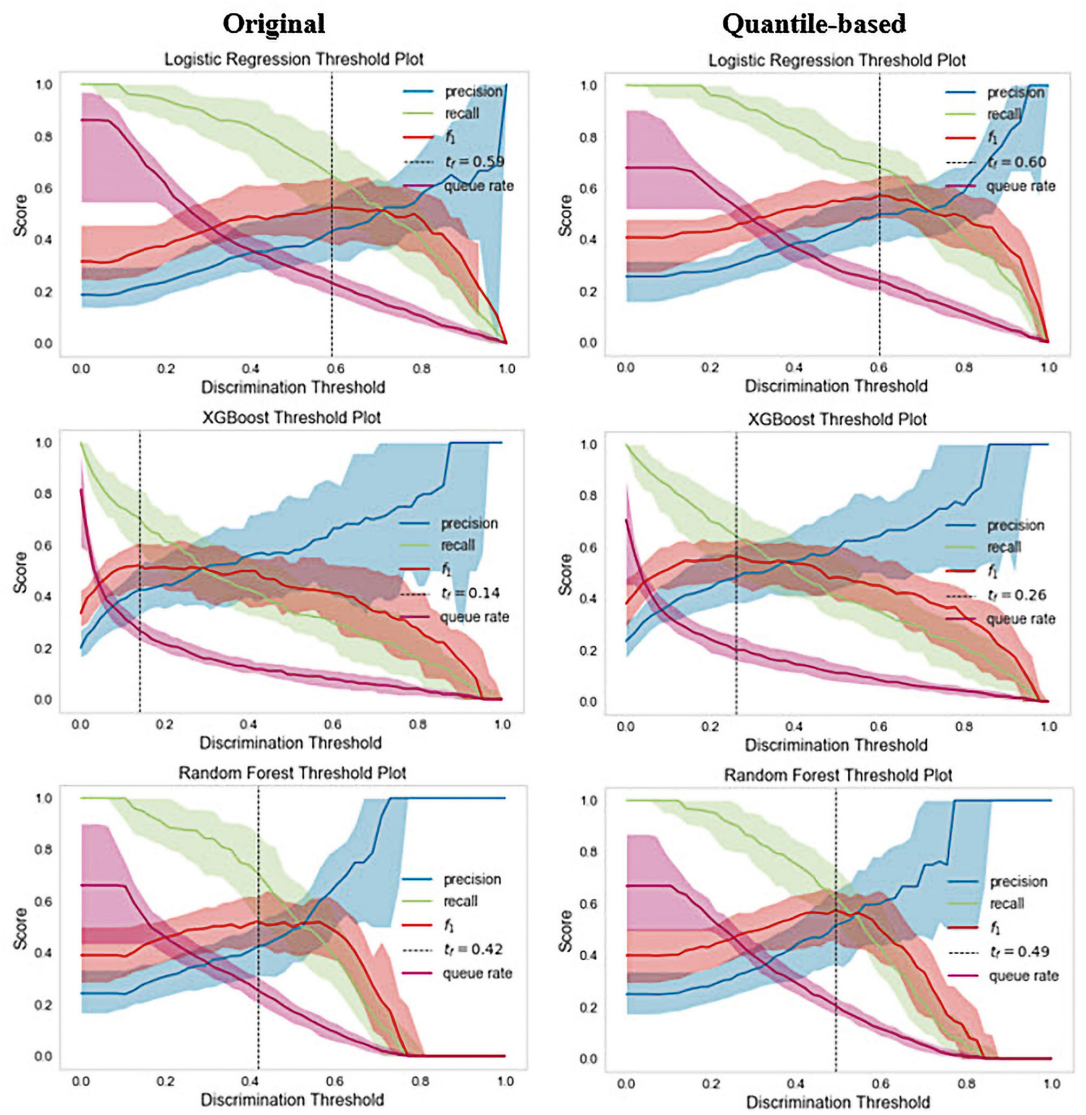
Interpolated precision-recall-F1 curve. The vertical dotted lines indicate the recall at which the curves achieve optimal precision.

To further investigate the depression detection model on the K-NHANES dataset, we compared the ML classifier discrimination threshold to differentiate between depression and non-depression cases by plotting the discrimination threshold using the package called Yellowbrick (67). The threshold plot is a plot of the various precision, recall, and F1 scores across different thresholds when predicting depression in the dataset. The plot supported the process of determining which threshold would be the optimal choice. A visualization of the precision, recall, and F1 score, concerning the classifier discrimination threshold, is shown in Figure 5. Generally, the DT plot is set at 50%; however, a comparison of the DT plot below and the original one shows that the optimal thresholds for LR, XGB, and RF were 59, 14, and 42%, respectively. However, for the quantile-based dataset, the optimal threshold was slightly higher than that of the original datasets; LR, XGB, and RF had thresholds of 60, 26, and 49%, respectively. Surprisingly, all models in the two datasets also boosted the same queue rate (~20%).

**Figure 6:**
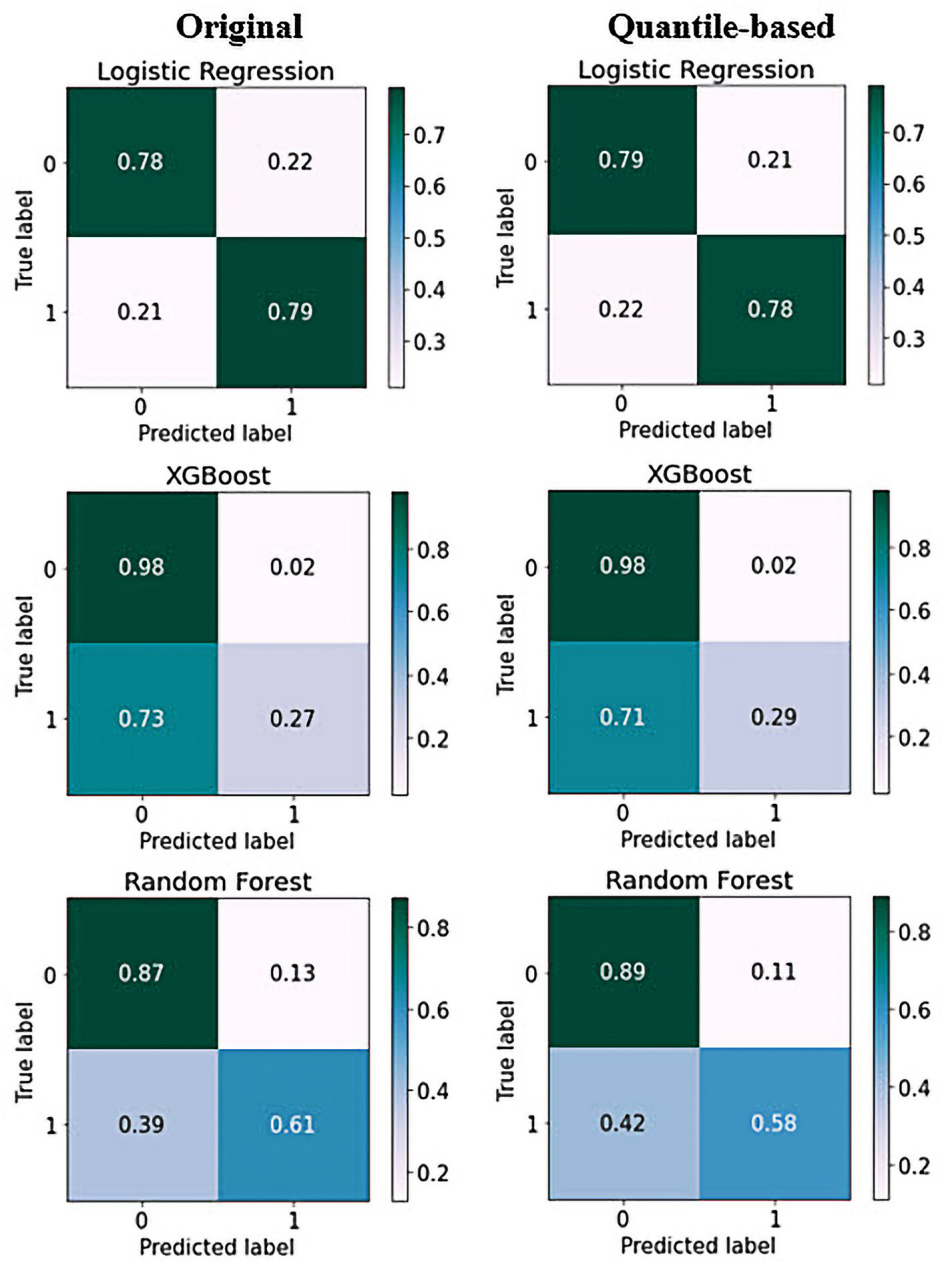
Confusion matrix of top three models on original and quantile-based datasets: Each of the confusion matrices are visualized as a color-coded heat map. It can be observed that all the plotted confusion matrices have darker cells for the diagonal elements. This indicates that more data are being predicted correctly to their respective label. Conversely, the off-diagonal elements with light shades indicate misclassifications done by the models.

To complement the results presented in this work, we provide details regarding the performance of the ML models in terms of the confusion matrices. Figure 6 shows the confusion matrices that allowed us to assess the performance of ML for the classification of depressive disorders. The horizontal axis represents the target class outcomes and the vertical axis represents the predicted class in the test dataset. XGBoost predicted the non-depression group with the highest accuracy and the depression group with the lowest accuracy when trained from scratch, and it correctly classified 98% of non-depression cases. In contrast, the depression group had the highest classification accuracy with LR achieved an excellent classification of both classes, with 78% in the non-depression group and 79% in the depression group. The results from the quantile-based dataset demonstrated a slight improvement in the non-depression group in the RF and LR classifiers.

**Figure 7:**
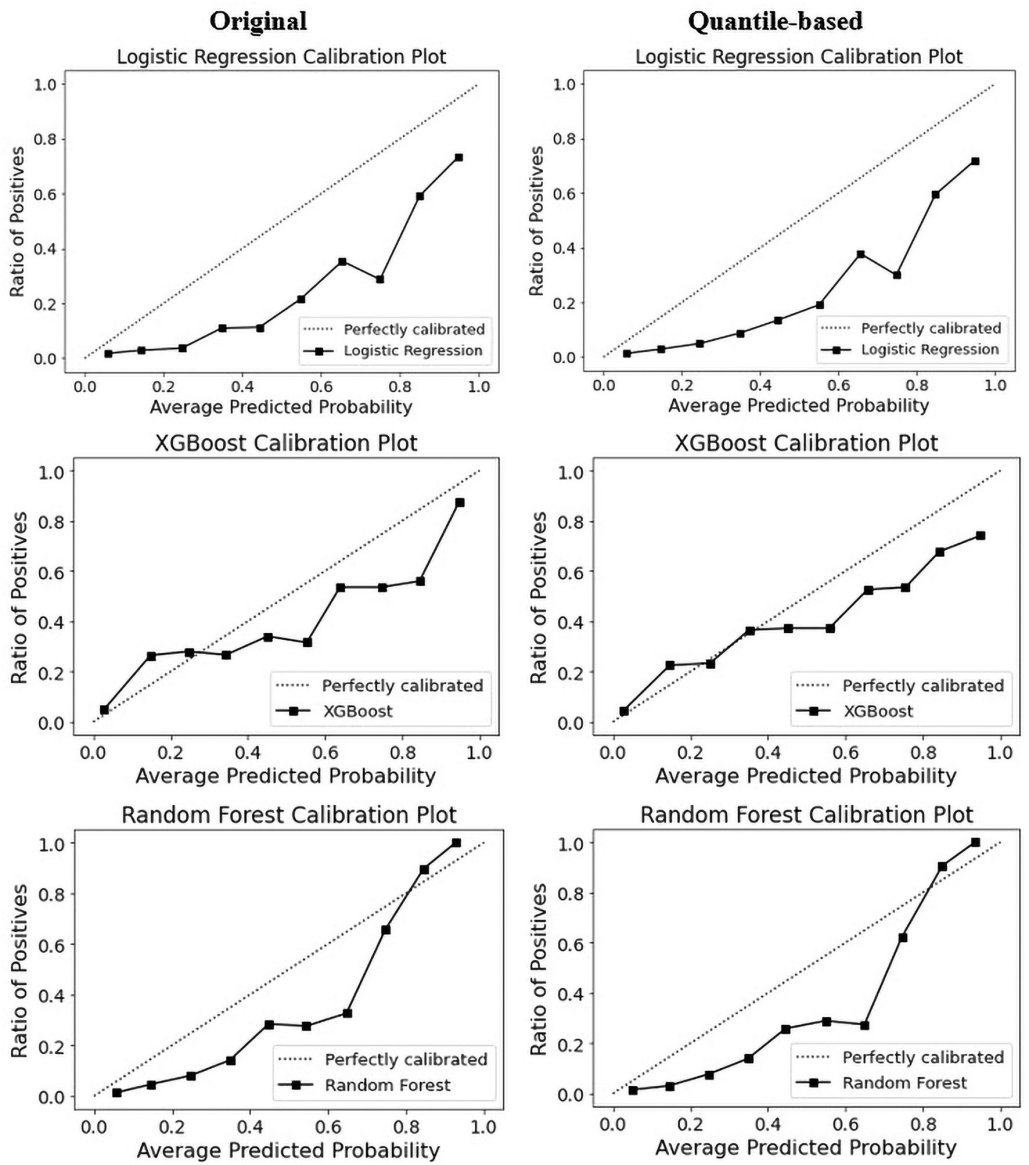
Calibration curve of top three models on original and quantile-based datasets.

The calibration curve is a linear relationship between the independent and dependent variables using a least squares method (68). The data are split into groups called “bins.” The y-axis shows the number of positive cases in each bin, while the x-axis shows the probability that the classifier predicted. The more closely the generated calibration curve approaches the standard line, the more closely the model's predictions align with the actual class distribution in the dataset. Assessing the calibration performance of risk prediction models based on machine learning algorithms receives attention in the medical field (69–71). Figure 7 shows the reliability curve of the classifier. The results revealed that the calibration slope from the models with the quantile-based dataset was closer to the intercept, which indicates a better predictive ability for the scaled version of the database. Regarding classifier comparison, XGB models are better calibrated than the RF and LR models for the original or quantile-based datasets.

### 3.4. Model explanation and interpretability of depression prediction

#### 3.4.1. Explanation of the model at the global level

Clinically, ML-based models seem promising; however, their interpretability has generally been overlooked. Recently, there has been a growing movement to make ML models more transparent, with a primary focus on opening-up black box algorithms based on *post hoc* model-agnostic methods, namely, feature importance, to enable the user to understand the model (72–76). Feature importance and visualization are important and widely used analysis methods in ML for calculating relative importance scores. By calculating the scores for each feature, clinicians can determine which features contribute the most to the predictive power of the model for disease diagnosis. It is mainly applied in clinical and biomedical areas because of the simplicity and interpretability of feature ranking and risk analysis (77–79). We performed permutation importance methods to evaluate variable ranking. The permutation importance score compares the significance of each characteristic to one that has been uniformly distributed, thereby eliminating the possibility of bias (80).

**Figure 8:**
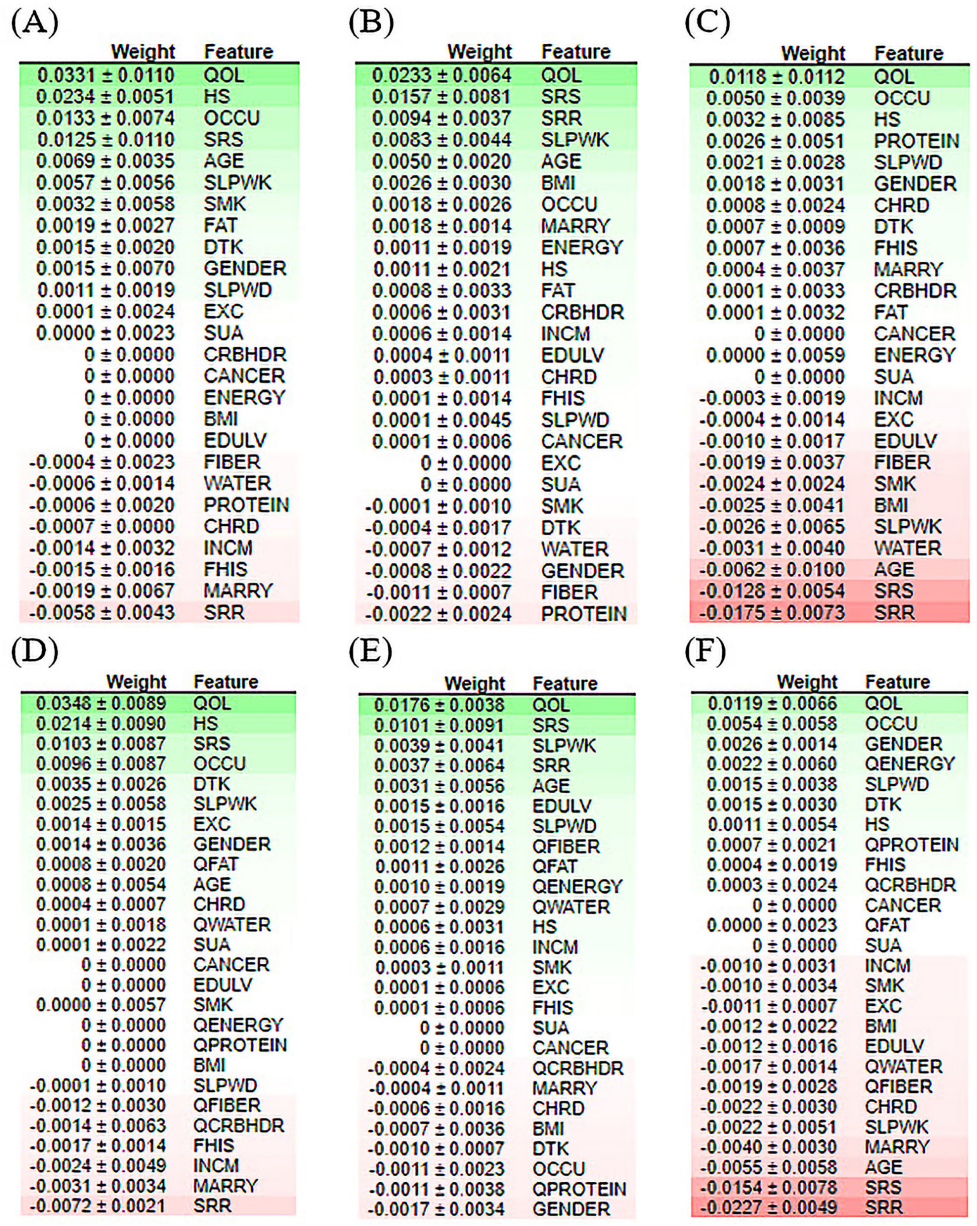
Feature contribution analysis performed by ELI5 for the classification of the depression model of the top three machine learning models. **(A)** LR model for the original dataset, **(B)** XGB model for the original dataset, **(C)** RF model for the original dataset, **(D)** LR model for the quantile-based dataset, **(E)** XGB model for the quantile-based dataset, and **(F)** RF model for the quantile-based dataset.

Additionally, this score is generated using test data, which provide a more accurate representation of the behavior of the model when responding to new input data. Recently, permutation-based feature importance has been introduced to the interpretability of machine-learning models (77). The importance of each feature is calculated using Eli5 (81). Eli5 is a Python library that obtains the global feature contribution toward the prediction. It works for regression and classification models and supports all Scikit-learning algorithms. As shown in Figure 8, quality of life achieved a higher rank for all ML models. Protein and fat, as nutritional indicators, appeared repeatedly at the top of the positive and negative rankings. This phenomenon confirmed that nutrition is a significant risk factor for depressive disorders. For the RF model, stress-related features had strong negative effects on depression prediction. For the XGB model, among the nutritional factors, carbohydrates demonstrated the lowest ranking for the prediction of depression. For both the RF or XGBoost model, BMI, QWATER, and sleep time revealed a robust negative ranking. Most importantly, the feature ranking between the quantile-based dataset and the original-based nutrition value dataset revealed different rankings. For the best-performing classifiers in terms of accuracy (XGB), marital status, gender, and occupation are the second, third, and fourth most important features, respectively; however, in the quantile-based nutrition dataset, this ranking was replaced by HS, QFiber, and QProtein. For the best-performing classifiers in terms of AUC (RF), occupation, protein, HS, and gender stand in place of the second, third, and fourth, respectively, while in the quantile version dataset, their places are changed to fourth, seventh, and eighth, respectively. These changes in variable ranking are also true for contributors with negative importance. In addition to the nutrition-based variables, some demographic and common risk factors also significantly contributed to depression prediction, such as marriage, sleeping time, gender, and smoking. Occupation and marital status were ranked as essential factors causing depression by most models, reflecting the relationship between them and depressive disorders.

**Figure 9:**
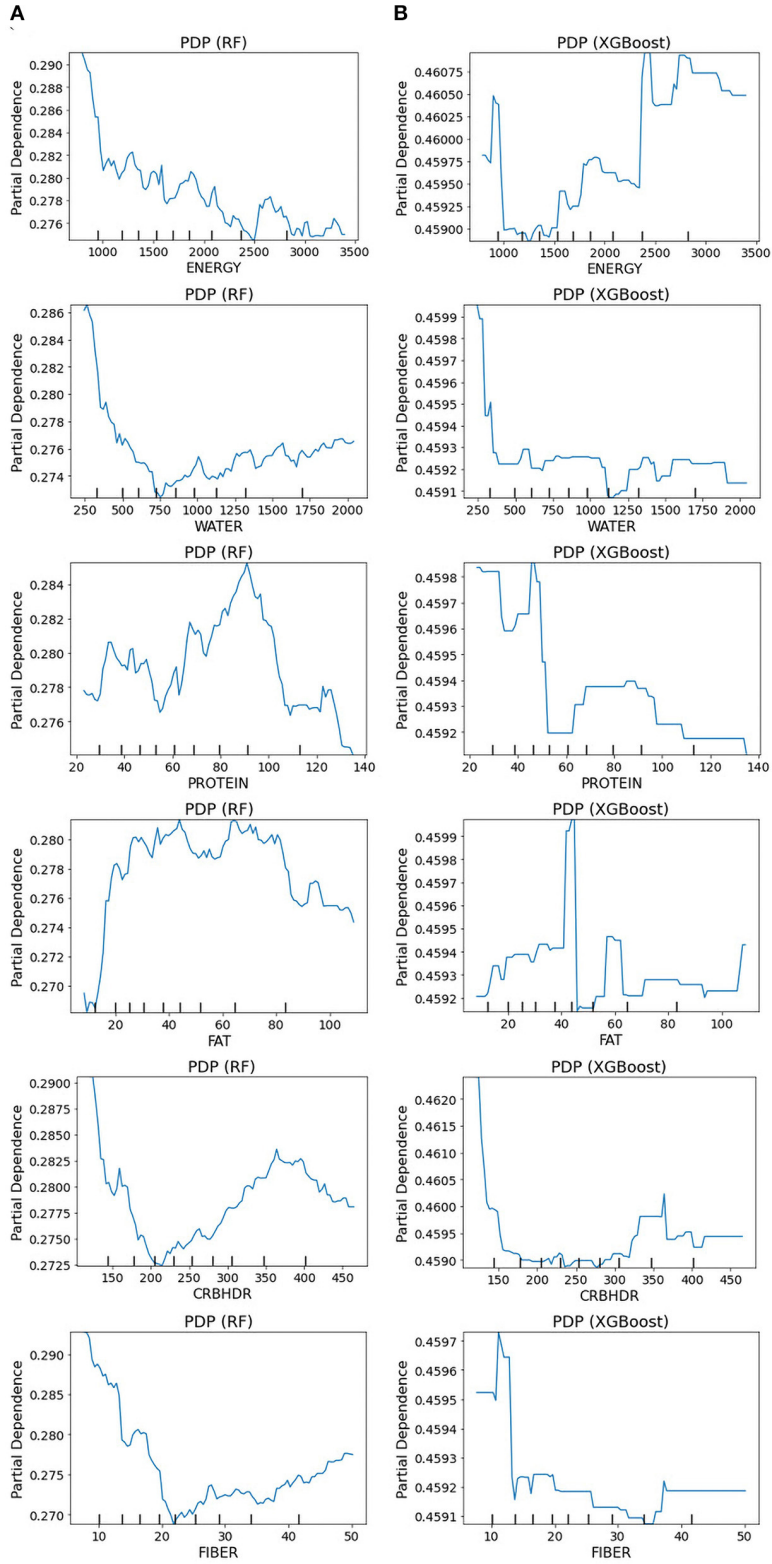
Partial dependence plots for the original dataset: **(A)** RF model and **(B)** XGBoost model. The partial plots show the dependencies of depression prediction change on each of the nutritional variables.

A partial dependency plot is another effective global interpretability method for understanding the relationship between variables and predictions. In ML, the marginal impact of one or two variables on the predicted outcome may be shown using a partial dependence plot (82). As illustrated in Figure 9, depression prediction decreased when the energy increased. The predicted depression tends to decrease with increasing energy values in the RF models. In contrast, in the XGBoost model, the expected depression tended to improve with an increase in the energy level. The RF and XGBoost models demonstrated the same trend in water consumption values for the final depression prediction. In the PD plot for proteins, the trend was no longer upward as the value increased from 100 in the RF model. However, in the XGBoost model, depression prediction was high, at 45, and a dramatic decrease was revealed afterward. Once the fat values became ~80, the prediction performance of RF improved; and height did not contribute to the overall age prediction (specified with the partial dependence of 0).

#### 3.4.2. Explanation of the model at the individual level

Explainable AI (XAI) has been a central focus of AI investigations owing to the critical need to establish algorithmic transparency. Explanation of the model at the individual level facilitates an understanding of how the model arrived at a particular prediction for a specific instance, which can improve its adoption and effectiveness in various fields. We next used LIME and SHAP force plots of nutritional features to illustrate their overall impact on the depression prediction model in individual patients.

LIME (83) is a local interpretation procedure that can be applied to any black-box ML model to provide a localized explanation for a single prediction. LIME is based on the simple but innovative principle of creating a new dataset of perturbed samples and retrieving the corresponding predictions from the black box model. LIME then assigns greater weight to altered samples and uses the weighted sample to construct an interpretable model. SHAP (SHapley Additive exPlanations) is a well-known framework that can be used for explaining the output of models at the individual level (84, 85).

**Figure 10:**
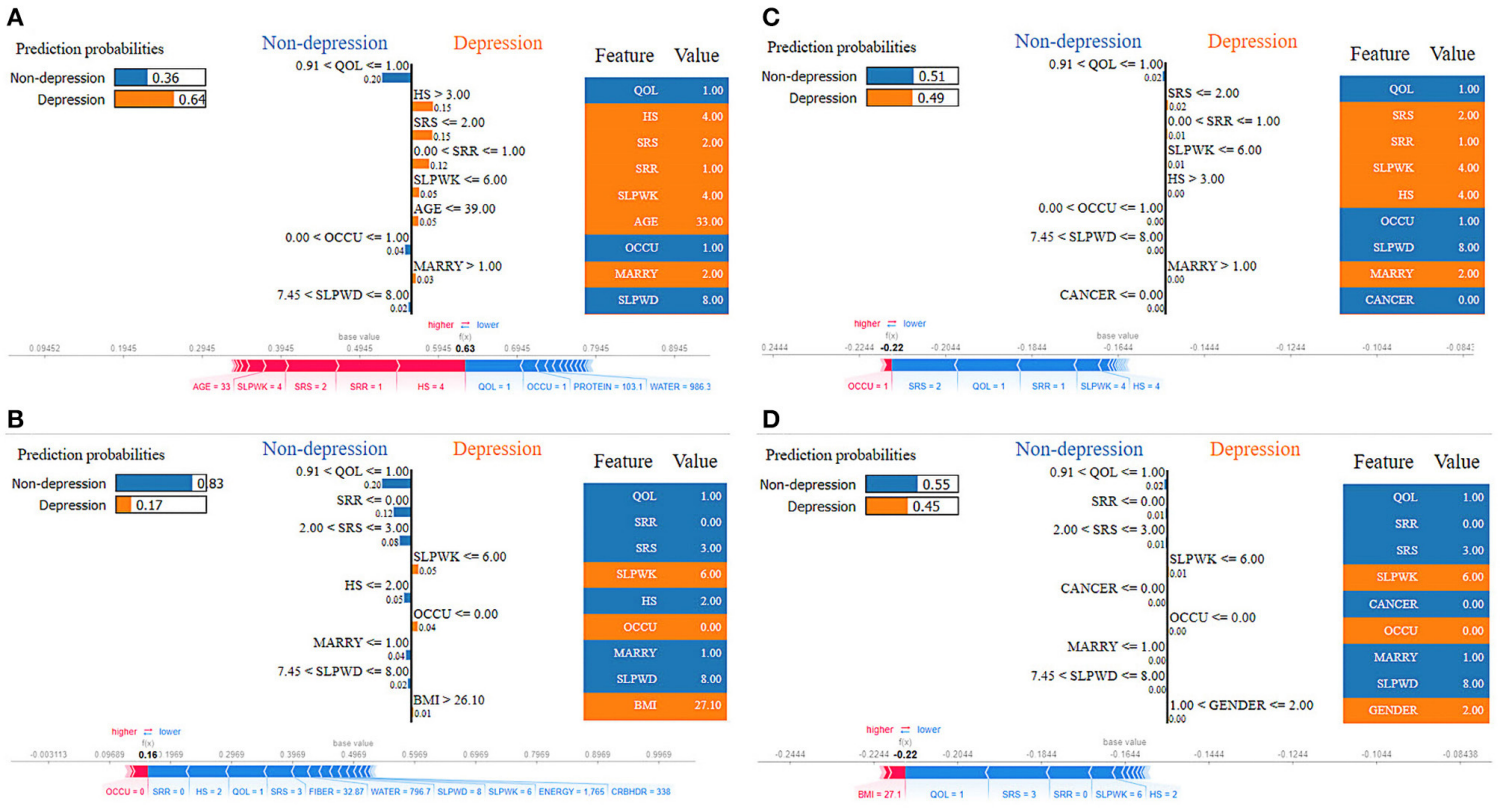
LIME and SHAP explanation plots of two representative individuals, patients 12 and 18. **(A)** Random forest model for an actual positive instance, **(B)** random forest model for an actual negative instance, **(C)** XGBoost model for an actual positive instance, and **(D)** XGBoost model for an actual negative instance.

As shown in Figure 10, the overall predicted probability of depression classification of two representative patients is illustrated in the LIME and SHAP plots. The RF model depression probability of patient 18 as an actual positive instance was reasonably high (0.64) due to positive conditions, including 0.91 <QOL ≤ 100, 0.00 <OCCU ≤ 1.00, 7.45 <SLPWD ≤ 8.00. The SHAP force plot illustrated similar findings of key features, plus protein and water (Figure 10A). In contrast, for patient 12, as an actual negative instance, the RF model predicted probability for non-depression was favorably high (0.83) due to many positive conditions: 0.91 <QOL ≤ 100, SRR ≤ 0.00, 0.00 <SRS ≤ 3.00, HS ≤ 2.00, MARRY ≤ 1.00, 7.45 <SLPWD ≤ 8.00. The SHAP force plot illustrated similar findings of key features, plus fiber, water energy, and carbohydrate (Figure 10B).

For the XGB classifier model, the depression probability of patient 18 as an actual positive case was relatively low, at only 51% due to the impact of variables including 0.91 <QOL ≤ 100, SRS ≤ 2.00, 0.00 <SRR ≤ 1.00, SLPWK ≤ 6.00, HS > 3.00, 0.00 <OCCU ≤ 1.00, 7.45 <SLPWD ≤ 8.00, MARRY > 1.00, and CANCER ≤ 0.00. The SHAP force plot illustrated similar findings of key features (Figure 10C). Conversely, for patient 12, as an actual negative instance, the XGB classifier predicted probability for non-depression at 55% with the impact of several key features, including 0.91 <QOL ≤ 100, SRR ≤ 0.00, 2.00 <SRS ≤ 3.00, SLPWK ≤ 6.00, CANCER ≤ 0.00, OCCU ≤ 0.00, MARRY ≤ 1.00, 7.45 <SLPWD ≤ 8.00, and 1.00 <GENDER ≤ 2.00. The SHAP force plot illustrated similar findings of key features plus BMI and HS (Figure 10D). These explanations at the individual level are consistent with those at the feature level and should further mitigate the black box concern.

## 4. Discussion

In summary, this study aimed to investigate the diagnostic ability of the ML-based algorithm in differentiating between individuals with depressive and non-depressive disorders. The evaluation of this ML study was based on a high-quality, large-scale EHR dataset from the K-NHANES database. Integrating a large-scale dataset to validate the proposed ML framework is of great importance in this research. This study presents an explainable ML method to aid clinical decision support enhanced by hyperparameter optimization in depression prediction (Figure 1). We evaluated the effectiveness of several ML algorithms for predicting depression outcomes based on a combination of 27 clinical and nutritional factors. Overall, the variables selected for the prediction model were the most clinically common and readily available, and showed efficient interpretability and consistency with clinical experience, further proving the reliability of the model. This also indicates that our model can be applied to countries and regions with relatively limited medical resources. Two original and quantile-based datasets were generated from the K-NHANES database. In the original dataset, we used the default values of all variables, whereas, in the quantile-based dataset, we converted the nutritional variable values to their quantile version. Normalization methods such as quantile normalization are crucial for high-dimensional data processing (86). Another finding of this study is the feasibility of quantile-based features that can be used to monitor decision-making patterns after implementing a classification and risk prediction model. For example, it was observed that there were only minor changes in learned decisions, suggesting that quantile-based features could even be extended to evaluate depression classification. The altered quantile-based dataset results also have potential feasibility for use by ML-based algorithms and could be transferred to other datasets and tasks.

Well-established visualization methods for interpreting high-dimensional depression data are scarce. To the best of our knowledge, this is the first study on ML to predict depression outcomes using nutritional and clinical data in an interpretable manner. We demonstrated generalizable applications of UMAP to provide informative insights into how an individual is distributed in the dataset. Visualizing the space of non-depression and depression data revealed substantial overlap, making classification a challenging task for ML classifiers. This may highlight the complexity of diagnosing depression (Figure 2). Correlations between variables were identified using Pearson correlation. The correlation between predictors and target variables was also investigated. The correlation coefficient of the 33 variables with depression also demonstrated the feasibility of ML to efficiently define complex relationships between multiple risk factor predictors and depression outcomes. Correlated variables are common in high-dimensional data. The correlation analysis in this study demonstrated that the generated datasets were free of collinearity and that the correlations between the variables were standard. Low-stress recognition with health status rate variables and self-recognized stress with quality-of-life variables were highly correlated (Supplementary Figure 1). All dietary variables were found to be negatively correlated with depression. The strongest correlation was found between depression and the fiber quantile, fiber, and protein (Figure 3). This is in line with the results of other reports that showed that low protein intake groups had a significantly higher risk of depression than the standard protein intake groups (60, 61). Several studies have demonstrated a potential relationship between depression and dietary fiber intake, in which an increased intake of total dietary fiber is associated with a lower likelihood of depression (63).

In this study, GridSearchCV was conducted to tune the hyperparameters of the classifiers, thus amplifying the classification performance. Although the use of the grid search with cross-validation technique requires higher computational costs, it is essential for evaluating the ML model performance. At the same time, there are risks of overfitting and problems with model fitness (87). Cross-validation is a fundamental method used to minimize overfitting and create better-fitting models (88). Several widely used classification measures, such as ACC, PREC, REC, AUC, ROC curve, and PRC curve, were generated to compare and visualize the performance of the algorithms. The classification results showed that the well-optimized approach achieved a promising performance. We found that the performance of XGBoost and RF in predicting depression was high in terms of accuracy and AUC, respectively, whereas a relatively low accuracy was observed for the SVM model (Table 1). Specifically, we evaluated our proposed method against a widely used baseline method on the same dataset and obtained a baseline score of 77%. Our proposed method achieved a significantly higher score of 85%, demonstrating the superiority of our approach.

We compared our results with two previous studies that achieved RCO values of 86% and AUC of 77%, respectively. Our study achieved an 86% ROC curve score for both the original dataset and the quantile-based dataset using the RF model, which is similar to the first study. Additionally, our study achieved an AUC value of 84.96% with the RF model on the original dataset and an 85.34% with the XGB model which is noticeably higher than the previous study. One important aspect of our study is that our datasets had a higher positive class ratio than the previous two studies. Specifically, our datasets had a positive class ratio of 16.09%, while the positive class ratios of the previous studies were 7 and 2%, respectively. This higher positive class ratio is an important finding as it suggests that our datasets had a higher prevalence of depression cases compared to the previous studies. Another important aspect of our study is that we performed a more detailed interpretable analysis, which can help in understanding how the machine learning models are making predictions. Specifically, we utilized feature importance analysis to identify the important nutritional factors that contribute to depression prediction. This analysis allowed us to identify which features were most predictive of depression and how they interacted with each other.

Given that RF and XGBoost showed similar performances, we think that fine-tuning might enable XGBoost to perform slightly better than RF. An AUC analysis is often insufficient for comparing predictive models, particularly in imbalanced datasets. Therefore, we used ROC and PRC curves to evaluate the performance of the five ML methods. Compared with other models, the RF model exhibited better performance, with a ROC curve of 86% for both datasets, and the XGBoost model exhibited a PRC of 49% in the original and 50% in the quantile-based datasets (Figure 4). LR demonstrated a lower misclassification for positive cases when the confusion matrix was examined. However, the XGB exhibited the worst performance (Figure 6). Although several high-quality models exist for EHR-based depression identification, most overlook interpretability, which is crucial for a model's credibility. Given that similar performances were found among LR, XGB, and RF in this retrospective cohort study, we further evaluated their calibration ability and decision threshold (Figures 5, 7).

To the best of our knowledge, this is the first study to use ML to predict depression outcomes using nutritional and clinical data in an interpretable manner. Considering the severe consequences that might result from a poor medical diagnosis, professionals have a solid inclination to cast doubt on AI models that fail to provide further explanations for their decisions. While clinicians use their clinical judgment when assessing patients, we believe that a predictive model could offer explanations for prognosis in a way that physicians can interpret. Thus, explainable AI algorithms have been developed for healthcare applications (89). This study attempted to provide the interpretability of ML models by presenting techniques for global and local interpretations. To address this issue, we introduced two widely used techniques, ELI5 and PDP for global interpretability, and LIME and SHAP for local interpretation. In general, both approaches have the potential to be equally valid depending on the application specifications. Global interpretability approaches have the benefit that they can be generalized over the entire population, whereas local interpretability techniques deliver explanations at the individual case level. In the feature importance analysis (Figure 8), the high-ranking features in predicting depression appeared to be the QOL among all classifiers.

Furthermore, our results showed that nutritional markers such as protein and fat consistently ranked highly in both positive and negative rankings. In contrast, carbohydrates had the lowest ranking in predicting depression. The most significant implication is that there is notable variability in the feature rankings between the quantile-based dataset and the original-based nutrient value dataset, which should be considered. The PDP plots further demonstrate the expected distribution of the impact of each feature in the superior models. With the help of PDP, the marginal effects of nutrition-selected features were efficiently analyzed. The PDP showed a threshold above which the probability of depression increased. The explainable results showed relative variations in the attribute values. For example, in the case of energy, the predicted depression tended to decrease in the RF models with increased energy values. In contrast, in the XGBoost model, the expected depression tended to improve with an increase in the energy level (Figure 9). Thus, we can conclude that interpretability is model-dependent and should be considered when selecting a model for prediction and classification tasks. Obtaining local explanations is another approach that employs local explainers to address application needs, and such a method requires considerable processing power. The LIME and SHAP technique provides a representation that is both local and interpretable by illustrating the relative differences between the variables that impact the depression prediction model in individual patients (Figure 10).

This study has some limitations. First, this was a single-race study, and external validation was required. Second, this was a retrospective study, and the findings should be validated in prospective studies. The strengths of this study are as follows: First, we used a large cohort dataset to build an ML model to predict depression. This could contribute to improving the practical training and explanation of the prediction model such that the model is closer to the actual situation of the prediction power. Second, we used hyperparameter optimization to build a prediction model to overcome the AI chasm, thereby improving its performance. Third, we used a variety of ML algorithms to select the optimal model that best fits the dataset. Finally, we generated a transformed dataset in which nutritional variables were converted into quantiles to facilitate rapid detection.

## 5. Conclusion

This research concentrates on developing a fully automated supervised ML-based classification system that can be used to classify depression using an interpretable approach. The complex nature of depressive disorders requires a large-scale study with an efficient source of features. The extraction and selection of nutritional features from a large-scale EHR-based system provided well-combined features indicative of diagnosing and monitoring depression. By doing so, well-fitted ML models can learn complex patterns from the interactions between risk factors and achieve a reasonable classification performance. Predicting the development of depression risk may require a global understanding of the fundamental risk factors for developing a depressive disorder. To provide a high-level explanation, our interpretable approach further identified more important risk factors that appear to be significant in driving the risk of depression. Various interpretability approaches may vary in their explanations of the performance of the ML model. Clinicians need to be familiar with the differences between ML-based interpretability methods and determine which works best for their specific questions. With additional work and validation, we believe that our prediction model could potentially contribute to an early depression diagnosis, leading to the development of more effective preventive measures.

## Code availability

The code used in this study is available from the corresponding author upon reasonable request (https://github.com/payam-kassani/Depression-and-Nutrition-a-Machine-Learning-study).

## Data availability statement

Publicly available datasets were analyzed in this study. This data can be found here: https://www.cdc.gov/nchs/nhanes/.

## Author contributions

PH: conceptualization of study, conceived and designed the methodology, carried out all the analysis and experiment methodology, and wrote the original draft. JL: data curation. J-WJ and CP: analysis and discussion of the results and investigation. C-HY: review and editing and investigation. S-AL: project administration, resources, funding acquisition, supervision, and review and editing. All authors have read and agreed to the published version of the manuscript.

## References

[1] Baldessarini RJ Forte A Selle V Sim K Tondo L Undurraga J. Morbidity in depressive disorders. Psychother Psychosom. (2017) 86:65–72. 10.1159/000448661 28183075

[2] Kessler RC Bromet EJ. The epidemiology of depression across cultures. Annu Rev Public Health. (2013) 34:119–38. 10.1146/annurev-publhealth-031912-114409 23514317 PMC4100461

[3] Mouchet-Mages S Baylé FJ. Sadness as an integral part of depression. Dialogues Clin Neurosci. (2008) 10:321–7. 10.31887/DCNS.2008.10.3/smmages 18979945 PMC3181878

[4] Nguyen DT Wright EP Dedding C Pham TT Bunders J. Low self-esteem and its association with anxiety, depression, and suicidal ideation in vietnamese secondary school students: a cross-sectional study. Front Psychiatry. (2019) 27:10. 10.3389/fpsyt.2019.00698 31611825 PMC6777005

[5] Layne C Merry J Christian J Ginn P. Motivational deficit in depression. Cognit Ther Res. (1982) 6:259–73. 10.1007/BF01173575

[6] Cipriani A Barbui C Geddes JR. Suicide, depression, and antidepressants. BMJ. (2005) 330:373–4. 10.1136/bmj.330.7488.373 15718515 PMC549094

[7] Zubrick SR Hafekost J Johnson SE Sawyer MG Patton G Lawrence D. The continuity and duration of depression and its relationship to non-suicidal self-harm and suicidal ideation and behavior in adolescents 12–17. J Affect Disord. (2017) 220:49–56. 10.1016/j.jad.2017.05.050 28595098

[8] Laursen TM Musliner KL Benros ME Vestergaard M Munk-Olsen T. Mortality and life expectancy in persons with severe unipolar depression. J Affect Disord. (2016) 193:203–7. 10.1016/j.jad.2015.12.067 26773921

[9] Noel PH. Depression and comorbid illness in elderly primary care patients: impact on multiple domains of health status and well-being. Ann Fam Med. (2004) 2:555–62. 10.1370/afm.143 15576541 PMC1466751

[10] GOLDBERG D. The aetiology of depression. Psychol Med. (2006) 36:1341–7. 10.1017/S0033291706007665 16740176

[11] Depression-Fact Sheets. Available online at: https://www.who.int/news-room/fact-sheets/detail/depression

[12] Global Depression Statistics. Available online at: www.sciencedaily.com/releases/2011/07/110725202240.htm

[13] Peter Heutink MR. The genetics of MDD – a review of challenges and opportunities. J Depress Anxiety. (2014) 3:2. 10.4172/2167-1044.1000150

[14] Flux MC Lowry CA. Finding intestinal fortitude: Integrating the microbiome into a holistic view of depression mechanisms, treatment, and resilience. Neurobiol Dis. (2020) 135:104578. 10.1016/j.nbd.2019.104578 31454550 PMC6995775

[15] Grajek M Krupa-Kotara K Białek-Dratwa A Sobczyk K Grot M Kowalski O. Nutrition and mental health: a review of current knowledge about the impact of diet on mental health. Front Nutr. (2022) 22:9. 10.3389/fnut.2022.943998 36071944 PMC9441951

[16] Ljungberg T Bondza E Lethin C. Evidence of the importance of dietary habits regarding depressive symptoms and depression. Int J Environ Res Public Health. (2020) 17:1616. 10.3390/ijerph17051616 32131552 PMC7084175

[17] Ma Y Li R Zhan W Huang X Zhang L Liu Z. The joint association between multiple dietary patterns and depressive symptoms in adults aged 55 and over in northern China. Front Nutr. (2022) 7:9. 10.3389/fnut.2022.849384 35330707 PMC8940515

[18] Ortega MA Fraile-Martínez Ó García-Montero C Alvarez-Mon MA Lahera G Monserrat J. Nutrition, epigenetics, and major depressive disorder: understanding the connection. Front Nutr. (2022) 18:9. 10.3389/fnut.2022.867150 35662945 PMC9158469

[19] Adjibade M Julia C Allès B Touvier M Lemogne C Srour B. Prospective association between ultra-processed food consumption and incident depressive symptoms in the French NutriNet-Santé cohort. BMC Med. (2019) 17:78. 10.1186/s12916-019-1312-y 30982472 PMC6463641

[20] Firth J Gangwisch JE Borsini A Wootton RE Mayer EA. Food and mood: how do diet and nutrition affect mental wellbeing? BMJ. (2020) 29:m2382. 10.1136/bmj.m2382 32601102 PMC7322666

[21] Carlson GA. The challenge of diagnosing depression in childhood and adolescence. J Affect Disord. (2000) 61:S3–8. 10.1016/S0165-0327(00)00283-4 11155966

[22] Rosalia RA Wahba K Milevska-Kostova N. How digital transformation can help achieve value-based healthcare: balkans as a case in point. Lancet Reg Heal - Eur. (2021) 4:100100. 10.1016/j.lanepe.2021.100100 34557815 PMC8454639

[23] Ostern N Perscheid G Reelitz C Moormann J. Keeping pace with the healthcare transformation: a literature review and research agenda for a new decade of health information systems research. Electron Mark. (2021) 31:901–21. 10.1007/s12525-021-00484-1 35599689 PMC8285287

[24] Yogesh MJ Karthikeyan J. Health Informatics: Engaging Modern Healthcare Units: A Brief Overview. Front Public Heal. (2022) 29:10. 10.3389/fpubh.2022.854688 35570921 PMC9099090

[25] Mollayeva T Sutton M Chan V Colantonio A Jana S Escobar M. Data mining to understand health status preceding traumatic brain injury. Sci Rep. (2019) 9:5574. 10.1038/s41598-019-41916-5 30944376 PMC6447542

[26] Kasani PH Kasani SH Kim Y Yun CH Choi SH Jang JW. An evaluation of machine learning classifiers for prediction of alzheimer's disease, mild cognitive impairment and normal cognition. In: International Conference on ICT Convergence. Jeju Island: IEEE (2021). p. 362–7.

[27] Meng W Sun Y Qian H Chen X Yu Q Abiyasi N. Computer-aided diagnosis evaluation of the correlation between magnetic resonance imaging with molecular subtypes in breast cancer. Front Oncol. (2021) 23:11. 10.3389/fonc.2021.693339 34249745 PMC8260834

[28] Win KY Choomchuay S Hamamoto K Raveesunthornkiat M Rangsirattanakul L Pongsawat S. Computer aided diagnosis system for detection of cancer cells on cytological pleural effusion images. Biomed Res Int. (2018) 2018:1–21. 10.1155/2018/6456724 30533436 PMC6250027

[29] Trinh N-HT Youn SJ Sousa J Regan S Bedoya CA Chang TE. Using electronic medical records to determine the diagnosis of clinical depression. Int J Med Inform. (2011) 80:533–40. 10.1016/j.ijmedinf.2011.03.014 21514880 PMC3124810

[30] Nam SM Peterson TA Seo KY Han HW Kang JI. Discovery of depression-associated factors from a nationwide population-based survey: epidemiological study using machine learning and network analysis. J Med Internet Res. (2021) 23:e27344. 10.2196/27344 34184998 PMC8277318

[31] Oh J Yun K Maoz U Kim T-S Chae J-H. Identifying depression in the National Health and Nutrition Examination Survey data using a deep learning algorithm. J Affect Disord. (2019) 257:623–31. 10.1016/j.jad.2019.06.034 31357159

[32] Greenland S. Invited commentary: variable selection versus shrinkage in the control of multiple confounders. Am J Epidemiol. (2007) 167:523–9. 10.1093/aje/kwm355 18227100

[33] Stoltzfus JC. Logistic regression: a brief primer. Acad Emerg Med. (2011) 18:1099–104. 10.1111/j.1553-2712.2011.01185.x 21996075

[34] Kweon S Kim Y Jang M-j Kim Y Kim K Choi S. Data resource profile: the korea national health and nutrition examination survey (KNHANES). Int J Epidemiol. (2014) 43:69–77. 10.1093/ije/dyt228 24585853 PMC3937975

[35] Singh D Singh B. Investigating the impact of data normalization on classification performance. Appl Soft Comput. (2020) 97:105524. 10.1016/j.asoc.2019.105524

[36] Arlot S Celisse A. A survey of cross-validation procedures for model selection. Stat Surv. (2010) 4. 10.1214/09-SS054

[37] Radmacher MD McShane LM Simon R A. Paradigm for class prediction using gene expression profiles. J Comput Biol. (2002) 9:505–11. 10.1089/106652702760138592 12162889

[38] Cox DR. The regression analysis of binary sequences. J R Stat Soc Ser B. (1958) 20:215–42. 10.1111/j.2517-6161.1958.tb00292.x

[39] Tin Kam Ho. Random decision forests. In: Proceedings of 3rd International Conference on Document Analysis and Recognition. Washington, DC: IEEE Computer Society Press (2002). p. 278–82.

[40] Webb GI Fürnkranz J Fürnkranz J Fürnkranz J Hinton G Sammut C. Decision tree. In: Encyclopedia of Machine Learning. Boston, MA: Springer US (2011). p. 263–7.

[41] Cortes C Vapnik V. Support-vector networks. Mach Learn. (1995) 20:273–97. 10.1007/BF00994018

[42] Chen T Guestrin C. XGBoost. In: Proceedings of the 22nd ACM SIGKDD International Conference on Knowledge Discovery and Data Mining. New York, NY: ACM (2016). p. 785–94.

[43] Tan P-N. Receiver operating characteristic. In: Encyclopedia of Database Systems. Boston, MA: Springer US (2009). p. 2349–52.

[44] Boyd K Eng KH Page CD. Area under the Precision-Recall Curve: Point Estimates and Confidence Intervals. New York, NY: SpringerLink (2013). p. 451–66.

[45] Van Rossum G Drake FL. Python 3 Reference Manual. Scotts Valley, CA: CreateSpace (2009).

[46] McKinney W O. Data structures for statistical computing in python. Proc 9th Python Sci Conf. (2010) 445: 51–6.

[47] Harris CR Millman KJ van der Walt SJ Gommers R Virtanen P Cournapeau D. Array programming with NumPy. Nature. (2020) 585:357–62. 10.1038/s41586-020-2649-2 32939066 PMC7759461

[48] Pedregosa F Varoquaux G Gramfort A Michel V Thirion B Grisel O. Scikit-learn: Machine learning in Python. J Mach Learn Res. (2011).

[49] Konopka BM Lwow F Owczarz M Łaczmański Ł. Exploratory data analysis of a clinical study group: development of a procedure for exploring multidimensional data. Batra SK, editor. PLoS ONE. (2018) 13:e0201950. 10.1371/journal.pone.0201950 30138442 PMC6107146

[50] Cook KA, Thomas, JJ,. Illuminating the Path: The Research Development Agenda for Visual Analytics. (2005). Available online at: https://www.osti.gov/biblio/912515

[51] McInnes L Healy J Melville J. UMAP: Uniform Manifold Approximation Projection for Dimension Reduction. (2018). Available online at: http://arxiv.org/abs/1802.03426

[52] Pearson's Correlation Coefficient. In: Encyclopedia of Public Health. Dordrecht: Springer Netherlands (2008). p. 1090–1.

[53] Zhu H Zhu Y Wu D Wang H Tian L Mao W. Correlation Coefficient Based Cluster Data Preprocessing and LSTM Prediction Model for Time Series Data in Large Aircraft Test Flights. New York, NY: SpringerLink (2018). p. 376–85.

[54] Li Y Horowitz MA Liu J Chew A Lan H Liu Q. Individual-level fatality prediction of COVID-19 patients using AI methods. Front Public Heal. (2020) 30:8. 10.3389/fpubh.2020.587937 33102426 PMC7556112

[55] Jaremka LM Lindgren ME Kiecolt-Glaser JK. Synergistic relationships among stress, depression, and troubled relationships: insights from psychoneuroimmunology. Depress Anxiety. (2013) 30:288–96. 10.1002/da.22078 23412999 PMC3816362

[56] Fluharty M Taylor AE Grabski M Munafò MR. The association of cigarette smoking with depression and anxiety: a systematic review. Nicotine Tob Res. (2017) 19:3–13. 10.1093/ntr/ntw140 27199385 PMC5157710

[57] Zhao L Han G Zhao Y Jin Y Ge T Yang W. Gender differences in depression: evidence from genetics. Front Genet. (2020) 15:11. 10.3389/fgene.2020.562316 33193645 PMC7593575

[58] Prasad S Sung B Aggarwal BB. Age-associated chronic diseases require age-old medicine: role of chronic inflammation. Prev Med. (2012) 54:S29–37. 10.1016/j.ypmed.2011.11.011 22178471 PMC3340492

[59] Maresova P Javanmardi E Barakovic S Barakovic Husic J Tomsone S Krejcar O. Consequences of chronic diseases and other limitations associated with old age – a scoping review. BMC Public Health. (2019) 19:1431. 10.1186/s12889-019-7762-5 31675997 PMC6823935

[60] Oh J Yun K Chae J-H Kim T-S. Association between macronutrients intake and depression in the United States and South Korea. Front Psychiatry. (2020) 17:11. 10.3389/fpsyt.2020.00207 32256414 PMC7090018

[61] Li Y Zhang C Li S Zhang D. Association between dietary protein intake and the risk of depressive symptoms in adults. Br J Nutr. (2020) 123:1290–301. 10.1017/S0007114520000562 32077385

[62] KHANNA P AERI BT. Association of quantity and quality of protein intake with depression and anxiety symptoms among adolescent boys and girls (13–15 years) studying in public schools of Delhi. J Nutr Sci Vitaminol. (2020) 66:S141–8. 10.3177/jnsv.66.S141 33612584

[63] Fatahi S Matin SS Sohouli MH Găman M-A Raee P Olang B. Association of dietary fiber and depression symptom: a systematic review and meta-analysis of observational studies. Complement Ther Med. (2021) 56:102621. 10.1016/j.ctim.2020.102621 33220451

[64] Kim C-S Byeon S Shin D-M. Sources of dietary fiber are differently associated with prevalence of depression. Nutrients. (2020) 12:2813. 10.3390/nu12092813 32937844 PMC7551178

[65] Mufti HN Hirsch GM Abidi SR Abidi SSR. Exploiting machine learning algorithms and methods for the prediction of agitated delirium after cardiac surgery: models development and validation study. JMIR Med Informatics. (2019) 7:e14993. 10.2196/14993 31558433 PMC6913743

[66] Saroj RK Yadav PK Singh R Chilyabanyama ON. Machine learning algorithms for understanding the determinants of under-five mortality. BioData Min. (2022) 15:20. 10.1186/s13040-022-00308-8 36153553 PMC9509654

[67] Bengfort B Bilbro R. Yellowbrick: Machine Learning Visualization. (2017). Available online at: http://www.scikit-yb.org/

[68] Moosavi SM Ghassabian S. Linearity of calibration curves for analytical methods: a review of criteria for assessment of method reliability. In: Calibration and Validation of Analytical Methods - A Sampling of Current Approaches. London: InTech (2018).

[69] Bolourani S Brenner M Wang P McGinn T Hirsch JS Barnaby D. A machine learning prediction model of respiratory failure within 48 hours of patient admission for COVID-19: model development and validation. J Med Internet Res. (2021) 23:e24246. 10.2196/24246 33476281 PMC7879728

[70] Lin X Lin S Cui X Zou D Jiang F Zhou J. Prediction-driven decision support for patients with mild stroke: a model based on machine learning algorithms. Front Neurol. (2021) 23:12. 10.3389/fneur.2021.761092 35002923 PMC8733999

[71] Gitto S Cuocolo R Annovazzi A Anelli V Acquasanta M Cincotta A. CT radiomics-based machine learning classification of atypical cartilaginous tumours and appendicular chondrosarcomas. EBioMedicine. (2021) 68:103407. 10.1016/j.ebiom.2021.103407 34051442 PMC8170113

[72] Le NQK. Explainable artificial intelligence for protein function prediction: a perspective view. Curr Bioinform. (2023) 20:18. 10.2174/1574893618666230220120449

[73] Vo TH Nguyen NTK Kha QH Le NQK. On the road to explainable AI in drug-drug interactions prediction: a systematic review. Comput Struct Biotechnol J. (2022) 20:2112–23. 10.1016/j.csbj.2022.04.021 35832629 PMC9092071

[74] Hung TNK Le NQK Le NH Van Tuan L Nguyen TP Thi C. An AI-based prediction model for drug-drug interactions in osteoporosis and paget's diseases from SMILES. Mol Inform. (2022) 41:2100264. 10.1002/minf.202100264 34989149

[75] Moss L Corsar D Shaw M Piper I Hawthorne C. Demystifying the black box: the importance of interpretability of predictive models in neurocritical care. Neurocrit Care. (2022) 37:185–91. 10.1007/s12028-022-01504-4 35523917 PMC9343258

[76] La CW Bauer C Moore JH Pendergrass SA. Interpretation of machine learning predictions for patient outcomes in electronic health records AMIA. Annu Symp Proc AMIA Symp. (2019) 2019:572–81. 32308851 PMC7153071

[77] Rodríguez-Pérez R Bajorath J. Feature importance correlation from machine learning indicates functional relationships between proteins and similar compound binding characteristics. Sci Rep. (2021) 11:14245. 10.1038/s41598-021-93771-y 34244588 PMC8270985

[78] Chung H Ko H Kang WS Kim KW Lee H Park C. Prediction and feature importance analysis for severity of COVID-19 in South Korea using artificial intelligence: model development and validation. J Med Internet Res. (2021) 23:e27060. 10.2196/27060 33764883 PMC8057199

[79] Thongprayoon C Jadlowiec CC Leeaphorn N Bruminhent J Acharya PC Acharya C. Feature importance of acute rejection among black kidney transplant recipients by utilizing random forest analysis: an analysis of the UNOS database. Medicines. (2021) 8:66. 10.3390/medicines8110066 34822363 PMC8621202

[80] Altmann A Toloşi L Sander O Lengauer T. Permutation importance: a corrected feature importance measure. Bioinformatics. (2010) 26:1340–7. 10.1093/bioinformatics/btq134 20385727

[81] Korobov M. LK. ELI5. (2016).

[82] Friedman JH. Greedy function approximation: a gradient boosting machine. Ann Stat. (2001) 29:1189–232. 10.1214/aos/1013203451

[83] Ribeiro MT Singh S Guestrin C. Why should i trust you? Explaining the Predictions of Any Classifier. (2016). Available online at: https://arxiv.org/abs/1602.04938 (accessed January 18, 2023).

[84] Ning Y Ong MEH Chakraborty B Goldstein BA Ting DSW Vaughan R. Shapley variable importance cloud for interpretable machine learning. Patterns. (2022) 3:100452. 10.1016/j.patter.2022.100452 35465224 PMC9023900

[85] Lundberg SM Erion G Chen H DeGrave A Prutkin JM Nair B. From local explanations to global understanding with explainable AI for trees. Nat Mach Intell. (2020) 2:56–67. 10.1038/s42256-019-0138-9 32607472 PMC7326367

[86] Hicks SC Okrah K Paulson JN Quackenbush J Irizarry RA Bravo HC. Smooth quantile normalization. Biostatistics. (2018) 19:185–98. 10.1093/biostatistics/kxx028 29036413 PMC5862355

[87] Greenwald HS Oertel CK. Future directions in machine learning. Front Robot AI. (2017) 24:3. 10.3389/frobt.2016.00079

[88] Montesinos López OA Montesinos López A Crossa J. Overfitting, model tuning, and evaluation of prediction performance. In: Multivariate Statistical Machine Learning Methods for Genomic Prediction. Cham: Springer International Publishing (2022). p. 109–39. 36103587

[89] Linardatos P Papastefanopoulos V Kotsiantis S. Explainable AI: a review of machine learning interpretability methods. Entropy. (2020) 23:18. 10.3390/e23010018 33375658 PMC7824368

