# Supplementary material for: Evaluation of nutritional status and clinical depression classification using an explainable machine learning method
Source: Front Nutr. 2023 May 9;10:1165854. doi: 10.3389/fnut.2023.1165854 (PMC10203418; doi:10.3389/fnut.2023.1165854)
Supplement: Supplementary file 1 [file Data_Sheet_1.docx]

Supplementary Material

Evaluation of Nutritional Status and Clinical Depression Classification Using an Explainable Machine Learning Method

Payam Hosseinzadeh Kasani, Jung Eun Lee, Chi Hyun Park, Cheol-Heui Yun, Jae-Won Jang, Sang-Ah Lee^*^

*** Correspondence:**Sang-Ah Lee
sangahlee@kangwon.ac.kr

# Supplementary Table

Supplementary Table 1. Hyperparameter settings for prediction models.

| **Algorithm** | **Optimal hyperparameter setting** |
| --- | --- |
| Logistic Regression | C = 1, penalty = 'l1', solver = 'saga', class_weight = {0: 0.6, 1: 3.1} |
| Random Forest | criterion= 'entropy', max_depth= 14, max_features= 'auto', max_samples= 0.3, min_samples_leaf= 6, min_samples_split= 4, n_estimators= 200, class_weight={0: 0.6, 1: 3.1} |
| Support vector machine | class_weight={0: 0.6, 1: 3.1}, C= 10, gamma= 'scale', kernel= 'rbf', probability=True |
| Decision Tree | class_weight={0: 0.6, 1: 3.1}, criterion= 'gini', max_depth= 6, max_features= 0.4, min_samples_leaf= 6, min_samples_split= 6, splitter= 'best' |
| XGBoost | class_weight={0: 0.6, 1: 3.1}, colsample_bytree= 0.5, gamma= 0.4, learning_rate= 0.3, max_depth= 4, reg_alpha= 1, reg_lambda= 1, verbosity = 0, silent=True, random_state=42 |

Supplementary Table 2. Statistical analysis for machine learning models

| Models Evaluation | One-way ANOVA | p-values |
| --- | --- | --- |
| **Original dataset** |  |  |
| Accuracy | 51.56 | p < 0.001 |
| Precision | 24.16 | p < 0.001 |
| Recall | 42.76 | p < 0.001 |
| F1 Score | 73.29 | p < 0.001 |
| AUC | 100.45 | p < 0.001 |
| **Quantile dataset** |  |  |
| Accuracy | 40.44 | p < 0.001 |
| Precision | 16.85 | p < 0.001 |
| Recall | 32.08 | p < 0.001 |
| F1 Score | 10.40 | p < 0.001 |
| AUC | 90.13 | p < 0.001 |

# Supplementary Figures

Supplementary Figure 1. Pairwise correlations of each feature measure across dataset. Cell values are Pearson’s correlation between pairs.

Supplementary Figure 2. Learning curve analysis based on number of samples using 5-fold cross-validation of top three models on original and quantile-based datasets.
