# Supplementary figures and images for: Evaluation of nutritional status and clinical depression classification using an explainable machine learning method
Source: Front Nutr. 2023 May 9;10:1165854. doi: 10.3389/fnut.2023.1165854 (PMC10203418; doi:10.3389/fnut.2023.1165854)

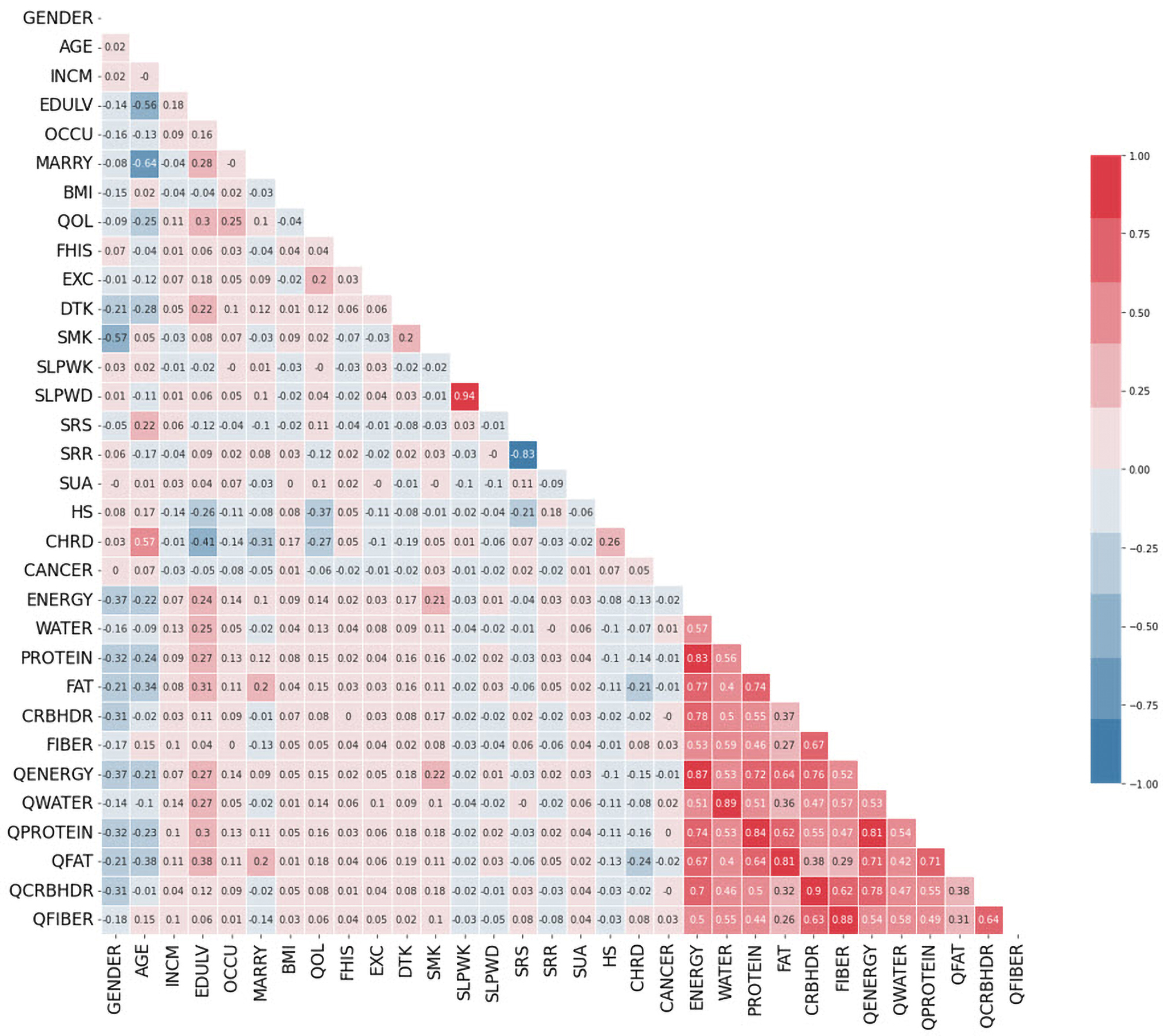

Supplement: Supplementary file 2 [file Image_1.JPEG]

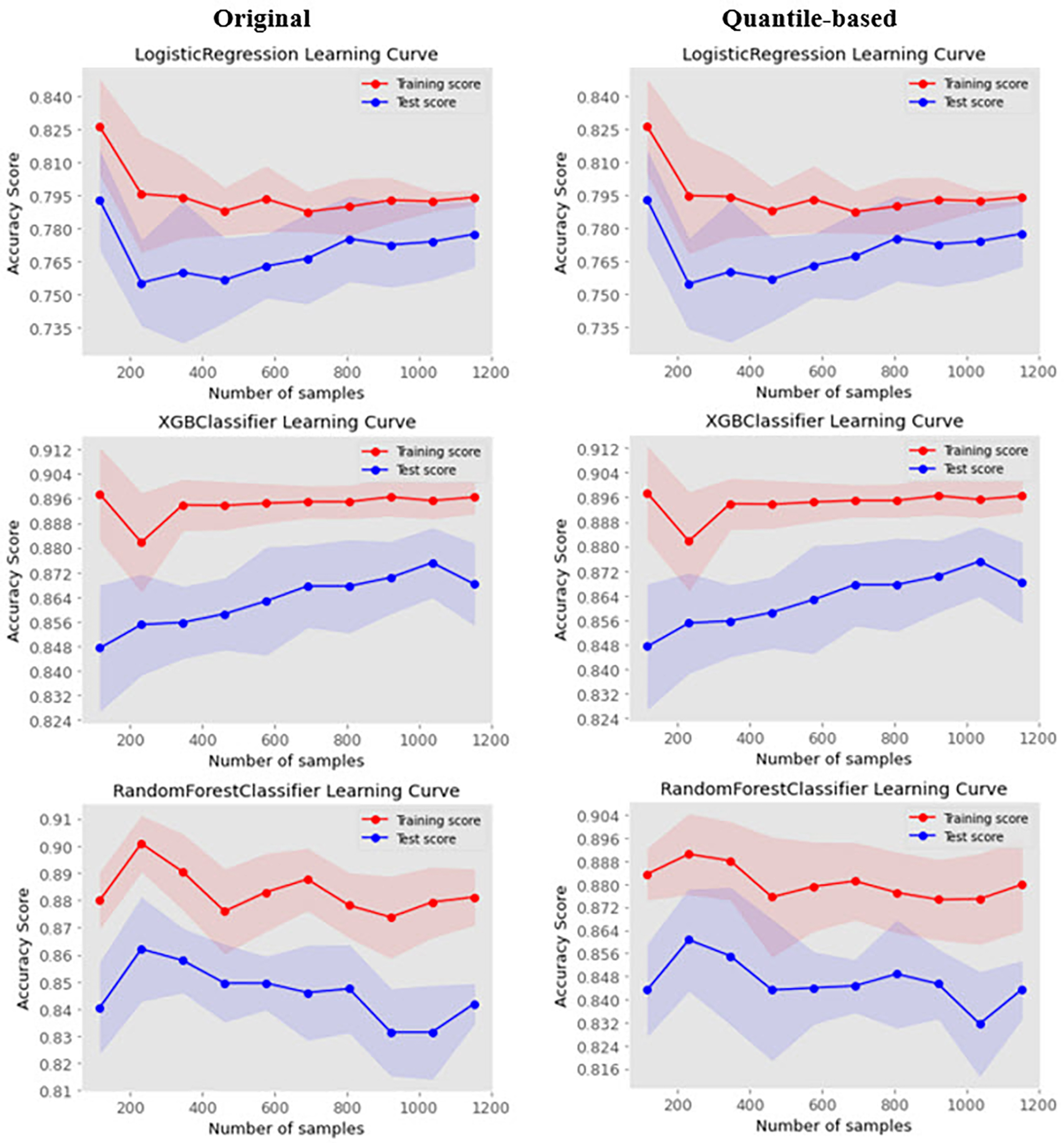

Supplement: Supplementary file 3 [file Image_2.JPEG]
